# Mitochondrial proteins encoded by the 22q11.2 neurodevelopmental locus regulate neural stem and progenitor cell proliferation

**DOI:** 10.1038/s41380-023-02272-z

**Published:** 2023-10-04

**Authors:** Philip D. Campbell, Isaiah Lee, Summer Thyme, Michael Granato

**Affiliations:** 1 Department of Psychiatry, Perelman School of Medicine, University of Pennsylvania, Philadelphia, PA 19104, USA.; 2 Department of Cell and Developmental Biology, Perelman School of Medicine, University of Pennsylvania, Philadelphia, PA 19104, USA.; 3 Department of Neurobiology, University of Alabama at Birmingham, Birmingham, AL 35294, USA.

## Abstract

Microdeletion of a 3Mb region encompassing 45 protein-coding genes at chromosome 22q11.2 (22q11.2DS) predisposes individuals to multiple neurodevelopmental disorders and is one of the greatest genetic risk factors for schizophrenia. Defective mitochondrial function has been hypothesized to contribute to 22q11.2DS pathogenesis; however, which of the six mitochondrial genes contribute to neurodevelopmental phenotypes and their underlying mechanisms remain unresolved. To systematically test 22q11.2DS genes for functional roles in neurodevelopment and behavior, we generated genetic mutants for each of the 37 conserved zebrafish orthologs and performed high throughput behavioral phenotyping using seven behavioral assays. Through this unbiased approach, we identified five single-gene mutants with partially overlapping behavioral phenotypes. Two of these genes, *mrpl40* and *prodha*, encode for mitochondrial proteins and, similar to what we observed in *mrpl40* and *prodha* mutants, pharmacologic inhibition of mitochondrial function during development results in microcephaly. Single mutant analysis shows that both *mrpl40* and *prodha* mutants display aberrant neural stem and progenitor cell proliferation, with each gene regulating distinct cell populations. Finally, double mutants for both *mrpl40* and *prodha* display aggravated behavioral phenotypes and neural stem and progenitor cell analysis reveals a previously unrecognized partially redundant role for *mrpl40* and *prodha* in regulating radial glia-like cell proliferation. Combined, our results demonstrate a critical role for mitochondrial function in neural stem and progenitor cell populations in the developing vertebrate brain and provide compelling evidence that mitochondrial dysfunction during neurodevelopment is linked to brain volume and behavioral phenotypes observed in models of 22q11.2DS.

## INTRODUCTION

Heterozygous microdeletion at chromosome 22q11.2 (22q11.2DS) is the most common microdeletion syndrome, occurring in 1 out of every ~2000 live births [1]. 22q11.2DS predisposes individuals to multiple neurodevelopmental disorders (NDDs), including schizophrenia (SZ), autism spectrum disorders (ASD), intellectual disability (ID), and attention-deficit/hyperactivity disorder (ADHD) [2]. Mouse models with similar heterozygous deletions display developmental deficits in neurogenesis [3], brain size [4], and ultimately circuit-level [5] as well as behavioral abnormalities [6], suggesting that this genetic locus has an integral role in early brain development that impacts postnatal behaviors and intellectual abilities. Thus, understanding the molecular mechanisms by which 22q11.2DS genes regulate brain development and behavior is fundamental to identifying therapeutic targets for individuals with 22q11.2DS and idiopathic forms of NDDs. Yet despite their importance, the neurodevelopmental and behavioral roles of all genes within the deleted region remain incompletely understood.

In the majority of cases, the deleted region encompasses 45 protein coding genes, of which ~90% are expressed in the brain in humans [7], suggesting that many genes within the region have the potential to contribute to neurodevelopmental phenotypes. While a subset of genes have been implicated in neuronal function and behavior, data on brain and/or behavioral phenotypes of individual mouse knockouts exists for only a fraction of the 45 genes (33%, 15/45) [8]. Even for those available, homozygous knockouts are frequently embryonic lethal, which precludes the analysis of functional roles these genes might have in behavior and brain function. Finally, a prevailing yet not fully tested hypothesis is that rather than single genes driving disease, interactions between genes in the region that overlap in function could be a driving factor [9], adding to the complexity of disentangling the functional contributions of the 45 genes to neurodevelopmental and behavioral phenotypes.

Recent work has suggested that genes with mitochondrial function may be one such driving factor [10–15]. Indeed, proteins encoded by at least six genes (13.3%, 6/45 genes) in the deleted region are localized to mitochondria [10] which represents an over two fold enrichment of mitochondrial genes in this region, given that roughly 5.7% of genes across the genome are localized to mitochondria (1136 of ~20,000 human genes [16]). Furthermore, neurons derived from individuals with the deletion display deficits in mitochondrial function [11] and patient phenotypic severity also appears to be correlated with deficits in mitochondrial function in patient cell lines [12]. At the same time, multiple lines of evidence over the last decade have implicated mitochondria in neurogenesis [17]. In fact, neurogenesis is affected in deletion mice [3] and individuals with 22q11.2DS display microcephaly, yet whether 22q11.2DS mitochondrial genes contribute to these phenotypes is an unresolved question.

To systematically test 22q11.2DS genes for their roles in neurodevelopment and behavior, we generated presumptive null mutants for all 37 conserved 22q11.2DS zebrafish orthologs. We then subjected these mutants to seven validated high-throughput larval behavioral assays that measure motor behavior, sensorimotor processing, habituation learning, and sensorimotor gating. This revealed five genes with partially overlapping behavioral phenotypes, suggesting the potential for roles in similar biological processes and/or circuits. Two of these genes, *mrpl40* and *prodha*, encode mitochondrial proteins, and similar to what we observe in these two mutants, pharmacologic inhibition of mitochondrial function leads to microcephaly. Moreover, we find that both *mrpl40* and *prodha* single mutants display phenotypes in neural stem and progenitor cell (NSPC) populations, though each mutant disrupts distinct NSPC populations; *mrpl40* mutants display prominent phenotypes in highly proliferative progenitors, whereas *prodha* mutants display phenotypes in less proliferative radial glia-like cells. Finally, we show that *mrpl40*/*prodha* double mutants display more severe behavioral phenotypes than single mutants and reveal an overlapping role for *mrpl40* and *prodha* in regulation of radial glia-like cell proliferation. Combined, these results identify two mitochondrial genes within the 22q11.2DS deleted region that play distinct, yet partially overlapping roles in regulating brain size and NSPC proliferation in vivo. Furthermore, our results suggest that in addition to deleterious effects in post-mitotic neurons, defective mitochondrial functioning plays a role during neurogenesis in 22q11.2DS-linked pathogenesis earlier than previously thought. Together, these results reveal a clear functional link between mitochondrial gene dysfunction, microcephaly, and 22q11.2DS-relevant behavioral phenotypes.

## RESULTS

### Generation of zebrafish 22q11.2DS orthologous gene mutants

**Fig. 1:**
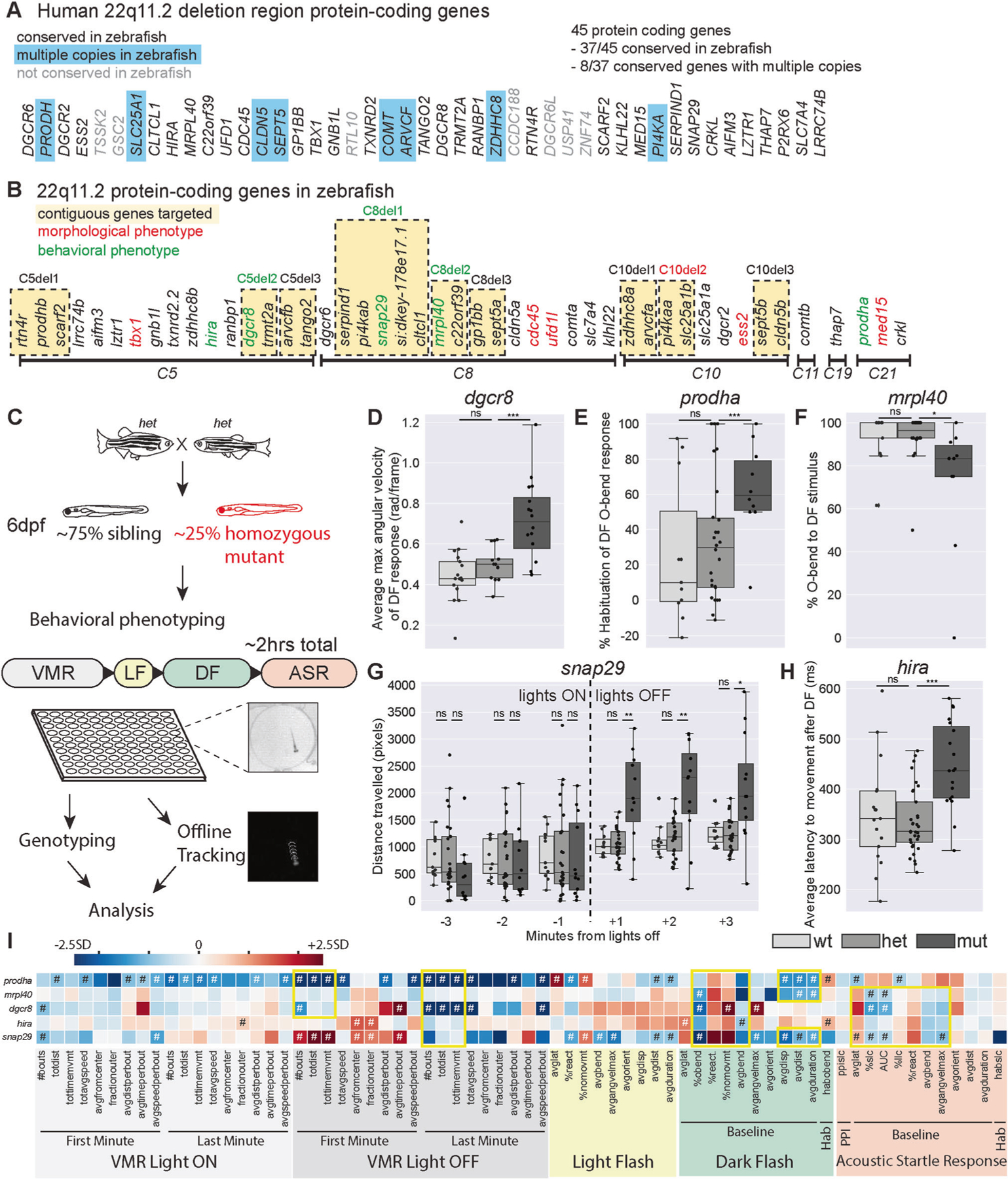
Mutants in five genes including two encoding mitochondrial proteins, display partially overlapping behavioral phenotypes. **A** Protein-coding genes present in the usual 3Mb human deletion. Those without orthologs in zebrafish are shaded gray. Those with multiple copies in zebrafish are highlighted in blue. **B** Zebrafish 22q11.2 orthologs are on six different chromosomes. Contiguous genes that were targeted for combination deletion are highlighted in yellow. Single or combination gene mutants with morphological or behavioral phenotypes are shown in red and green font respectively. **C** Behavioral phenotyping paradigm. Heterozygous carriers are in-crossed to generate homozygous mutants and siblings that are raised to 6dpf at which point behavioral phenotyping is performed blinded to genotype. Approximately 50 larvae are used for each mutant line, and arrayed in a 100-well plate. Assay occurs over approximately 2 h in the following order: Visual-motor Response (VMR), Light Flash (LF), Dark Flash (DF), Acoustic Startle Response (ASR). Individual larvae are genotyped, acquired videos are tracked offline, and then analyzed for 94 behavioral metrics. **D–H** Example phenotypes for five single gene mutants with reproducible behavioral phenotypes. Each plot represents data from a single behavioral run. Points indicate individual larvae, Boxes indicate range of 25^th^ to 75^th^ percentile with line indicating the median, whiskers indicate the rest of the range, with outliers lying outside of this. **D** Average maximum angular velocity of the DF response is increased in *dgcr8* mutants compared to siblings. **E** Percent habituation of DF O-bend response is increased in *prodha* mutants compared to siblings. **F** Percent of time *mrpl40* mutants perform O-bends in response to DF is decreased compared to siblings. **G** *snap29* mutants display an increase in distance traveled in VMR assay following lights turning off compared to siblings. Each bin represents the total distance traveled in during that minute of the assay. **H** Average latency to movement after DF stimulus is increased in *hira* mutants compared to siblings. All statistics represent one-way ANOVA, *p < 0.05, **p < 0.01, ***p < 0.001. I Heatmap illustrating single gene mutant phenotypes across behavioral metrics. Each row is an individual mutant line and all columns represent a behavioral metric. Color of each box represents difference of average mutant value compared to siblings expressed as number of standard deviations (SD). Blue boxes indicate the value is lower in the mutant and Red boxes indicate the value is higher in the mutant. Mutant values that are significantly different from siblings based on Student’s *t* test with a Bonferroni-corrected *p*-value of <0.05/94 are designated with #. Yellow boxes indicate behavioral similarities across mutant lines. VMR behavioral metrics are highlighted in gray, LF in yellow, DF in green, and ASR in orange. Supplementary Tables 5–7 display behavioral data and sample sizes for mutant lines with reproducible behavioral phenotypes. Python tracking scripts are in Supplementary Zip file 1.

To identify the protein-coding genes in the 22q11.2DS deleted region that play critical roles in neurodevelopment and behavior in vivo, we used the zebrafish model system. We previously reported that 37 of the 45 protein-coding genes in the 22q11.2DS deleted region have orthologs in zebrafish [18], with seven having duplicate copies and one having triplicate copies for a total of 46 zebrafish 22q11.2DS orthologous genes (Fig. 1A, B). To generate presumptive null alleles for each of the individual 46 genes, we used a CRISPR-Cas9 targeting approach with two guide RNAs (gRNA) targeting regions ~50–400 bp apart and flanking either a known functional domain or a highly conserved region (Supplementary Table S1). Using this approach, we identified heterozygous F2 adults with a variety of insertions and deletions (Supplementary Table S1). Mutations were predicted to lead to frameshifts (30/46, 65%), in-frame insertions/deletions (11/46, 24%), or disruption of splice sites (5/46, 11%) (Supplementary Table S1). Five of the 46 mutant lines displayed gross morphological defects in the homozygous state at 6 days post-fertilization (6dpf) (Fig. 1B, Supplementary Table S2), and these homozygous mutants were excluded from subsequent behavioral analyses. To assess behavior, we used a behavioral analysis pipeline that consisted of seven previously validated assays (described in detail in the following section). This pipeline identified five mutant lines that displayed behavioral defects at 6dpf (Fig. 1B, I). Importantly, these mutants did not display overt morphological defects, though two (*dgcr8* and *snap29*) had deficits in swim bladder inflation that were incompletely penetrant (Supplementary Table S3). Thus, our single gene mutational analysis identified 10 genes with roles in either development or behavior prior to 6dpf.

### Mutants in five genes including two encoding mitochondrial proteins, display partially overlapping behavioral phenotypes

As a first approach to identify genes with roles in brain development and/or function, we subjected all single-gene mutant lines to behavioral phenotyping at 6dpf (Fig. 1C). We used an unbiased phenotyping approach, assaying behavioral responses to visual and acoustic stimuli. Specifically, using visual stimuli we assayed the visual motor response (VMR) [19], the response to flashes of light (Light Flash, LF) [20], the response to flashes of darkness (Dark Flash, DF) [20], and Short-term habituation (Hab) of the DF response [21]. Using acoustic stimuli, we assayed the acoustic startle response (ASR) [22], as well as ASR modulation, including startle sensitivity [23], pre-pulse inhibition (PPI) [24] and startle habituation [21]. Behaviors of individual larvae were tracked at millisecond resolution, and all larvae were genotyped following completion of the behavioral assay (Fig. 1C). For each behavioral assay multiple kinematic parameters were computed using custom-written tracking software. Specifically, for the VMR assay, frequency, features of movement (speed, distance traveled, etc.), and position metrics were computed. For the LF, DF, and ASR assays, frequency of response to stimulus and features of the responses (bend angle, latency, distance, etc.) were computed. Homozygous mutant larvae were compared to sibling larvae across all behavioral metrics. In cases where gross morphological defects were observed in homozygotes, heterozygotes were compared to wildtypes.

Using this pipeline, we identified five single-gene mutants with reproducible behavioral phenotypes. Each of these mutant lines had behavioral phenotypes that spanned more than one assay. Further, phenotypes were undetectable in heterozygotes which appeared indistinguishable from wild-type siblings. Examples of phenotypes are displayed in Fig. 1D–H. Behavioral heatmaps revealed that phenotypes were specific both within each mutant line and between mutant lines (Fig. 1I). That is, not all behavioral metrics were affected in individual lines and each mutant line had a distinct subset of metrics affected. Closer examination and comparison of behavioral heatmaps revealed several overlapping phenotypes across mutant lines (Fig. 1I). All five mutant lines displayed abnormal baseline metrics for the DF response, indicating that these genes have roles in mediating sensorimotor behaviors in response to darkness. Three of the five lines (*mrpl40, dgcr8, snap29*) also displayed reductions in the sensitivity of the ASR response, while the fifth line (*prodha*) displayed differences in the opposite direction indicating these genes play roles in the hindbrain startle circuit or in downstream effectors. In addition to shared phenotypes, we also observed phenotypes unique to one or two mutant lines. For example, *snap29* mutants had increased movement metrics during the first minute of the VMR following lights turning off (Fig. 1G), *prodha* (Fig. 1E) and *hira* mutants had increased habituation of the DF response, and *dgcr8* mutants displayed increased angular velocity of the DF response (Fig. 1D). Regarding the biological function of these genes, there are also both similarities and differences: *prodha* and *mrpl40* have roles in mitochondria, *dgrc8* and *hira* have roles in gene regulation, while *snap29* is involved in vesicle trafficking. In summary, our systematic analysis of the 22q11.2DS deleted region identified five single genes that function to either establish or maintain several distinct and overlapping behaviors.

One hypothesis for how the 22q11.2DS deleted region generates disease risk is that genes within the region are involved in overlapping biological processes [9] resulting in partial functional redundancy, such that inactivating individual genes is insufficient to reveal measurable phenotypes. To assess whether the combined loss of multiple 22q11.2DS genes results in behavioral phenotypes, we combined multiple gRNAs to generate nine combination gene mutants that disrupted between 2 and 5 neighboring genes (Fig. 1B). This yielded deletions ranging in size from ~7kb-544kb (Supplementary Table S4). One of these deletions, *C10del2*, encompassed two genes, *pi4kaa* and *slc25a1b*, and resulted at 6dpf in gross morphological defects not present in single deletion mutants of either of the two genes (Fig. 1B, Supplementary Table S2), indicating that *pi4kaa* and *slc25a1b* may have partially overlapping function during early development. We then analyzed behavior of combination mutant lines. While three of the nine combination mutant lines had reproducible behavioral phenotypes (*C5del2*, *C8del1*, *C8del2*), the phenotypes were also detectable in single-gene mutants in genes present in the deleted region of the combination lines (*dgcr8*, *snap29*, *mrpl40* respectively) (Fig. 1B). Comparing behavioral heatmaps of these combination mutants with their corresponding single mutants revealed similar phenotypes (Supplementary Fig. S1), indicating that the loss of a single gene is driving phenotypes in these combination mutant lines. Although we only tested a limited number of multiple gene deletion combinations, our results suggest that directly neighboring 22qDS zebrafish orthologs exhibit only limited behavioral functional redundancy.

### Pharmacologic inhibition of mitochondrial function phenocopies the *mrpl40* mutant phenotype

**Fig. 2:**
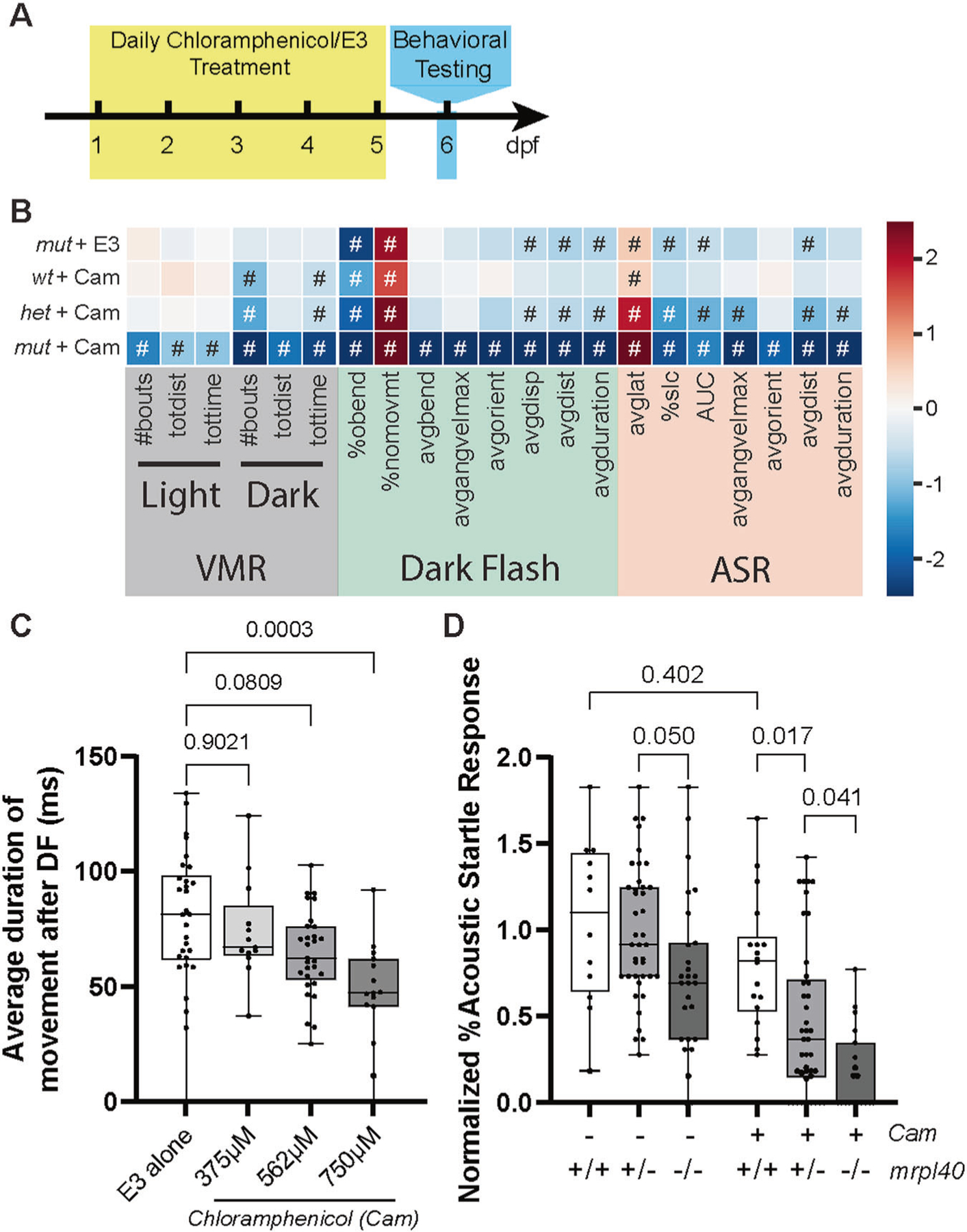
Pharmacologic inhibition of mitochondrial function phenocopies the *mrpl40* mutant phenotype. **A** Chloramphenicol experimental setup. Animals were treated daily with chloramphenicol in E3 or with E3 alone and then behaviorally tested at 6dpf. **B** Heatmap illustrating behavioral phenotype across VMR, DF, and ASR behavioral metrics based on *mrpl40* genotype and Chloramphenicol (Cam) treatment status. mut+E3 (*n* = 27), wt+Cam (*n* = 18), het+Cam (*n* = 41), mut+Cam (*n* = 19) from two biological replicates. Values that are significantly different from sibling/untreated controls based on Student’s *t* test with a *p*-value of <0.05 are designated with #. See Fig. 1 legend for full description of heatmap. **C** Chloramphenicol-treated larvae have dose-dependent reductions in the average duration of movement following DF stimuli. E3 alone (*n* = 30), 375 μM (*n* = 13), 562 μM (*n* = 29), 750 μM (*n* = 15) from three biological replicates. One-way ANOVA, *p*-values are displayed on the plot. **D** Percent Acoustic Startle Response based on *mrpl40* genotype and Cam treatment status. *mrpl40* heterozygotes and mutants are hypersensitive to Cam-treatment. Wildtypes (*n* = 12), hetererozygotes (*n* = 40), mutants (*n* = 27) with E3. Wildtypes (*n* = 18), hetererozygotes (*n* = 41), mutants (*n* = 19) with Chloramphenicol 562 μM from two biological replicates.. One-way ANOVA, *p*-values are displayed on the plot.

Two of the five genes identified through our behavioral screening (*mrpl40* and *prodha*) encode for proteins that localize to mitochondria [10], and defective mitochondrial function has been previously reported in the context of 22q11.2DS [11] as well as in neurodevelopmental disorders associated with 22q11.2DS [25]. As such, we hypothesized that disrupted mitochondrial functioning might contribute to the behavioral phenotypes we observed in *mrpl40* and *prodha* mutants. *mrpl40* is a component of the mitochondrial ribosome, and thus its disruption would be expected to decrease mitochondrial translation and subsequently reduce protein levels of electron transport chain components. To test whether inhibiting mitochondrial translation affects behavior and recapitulates the *mrpl40* phenotype, we treated wild-type embryos between 1 and 5 dpf with the mitochondrial ribosome inhibitor chloramphenicol (Cam) [26, 27] and tested their behavior at 6dpf (Fig. 2A). Compared to embryos reared in the absence of Cam, Cam-treated larvae displayed a dose-dependent behavioral profile that phenocopied *mrpl40* mutants (Fig. 2B, C). Specifically, Cam treatment recapitulates both DF and ASR phenotypes observed in *mrpl40* mutants (Fig. 2B).

*mrpl40* is maternally expressed [28] and part of a large multiprotein and RNA complex [29], supporting the idea that in *mrpl40* mutants mitochondrial translation may be reduced, rather than eliminated. Similarly, zebrafish embryos treated with Cam have been shown to have reduced, rather than completely absent electron transport chain complex activity, even at Cam doses higher than those used in our experiments [27]. Therefore, we reasoned that loss of *mrpl40* would render animals particularly sensitive to Cam treatment. Indeed, we find that animals heterozygous for *mrpl40*, which do not have phenotypes at baseline (Fig. 2D), are more sensitive to Cam treatment than wild-type animals, displaying more severe phenotypes and nearly fully recapitulating the *mrpl40* mutant phenotype (Fig. 2B, D). Furthermore, *mrpl40* mutants are extremely sensitive to Cam treatment, displaying phenotypes across all behavioral metrics (Fig. 2D). Together, this supports the hypothesis that mitochondrial *mrpl40* function is critical for distinct behaviors, including sensorimotor behaviors, in response to darkness and acoustic stimuli.

**Fig. 3:**
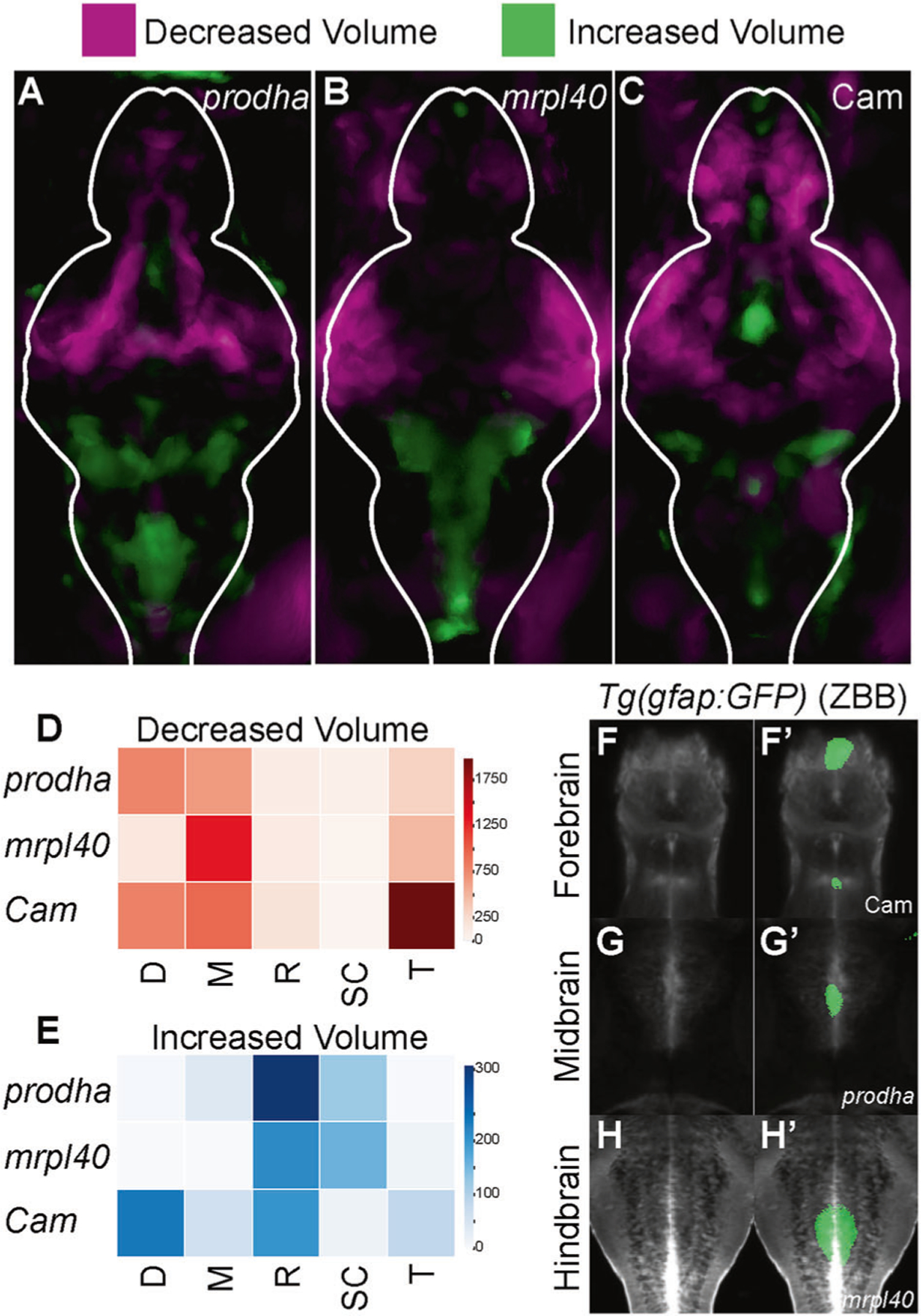
Mitochondrial disruption leads to alterations in brain volume. **A–C** Differences in brain volume in mitochondrial mutants and pharmacologically inhibited larvae. Deformation-based morphometry for *prodha* mutants (**A**), *mrpl40* mutants (**B**), and Chloramphenicol-treated (**C**) larvae shows differences in multiple brain areas. Images are sum projections of whole-brain z-stacks. Magenta designates areas that are reduced in volume compared to siblings/controls and Green designates areas that are increased in volume compared to siblings/controls. White outline indicates outline of brain. Analysis performed as in Thyme et al. [35]. Representative images of two biological replicates with at least *n* = 11 wildtypes/controls and *n* = 12 mutants/treated. **D–E** Heatmaps illustrating degree to which volume signals in **A–C** occupy various brain regions in arbitrary units. Higher values indicate larger reductions (**D**)/increases (E) in volume in designated region. Values are scaled based on size of the region as performed in Randlett et al. [36]. D diencephalon, M mesencephalon, R rhombencephalon, SC spinal cord, T telencephalon. **F–H’** Colocalization of increases in brain volume with *gfap* reporter line. Average signal of a *Tg(gfap:GFP)* line that has been registered to the Zebrafish Brain Browser (ZBB) atlas was merged with increased volume signals for *prodha* mutants (**G-G’**), *mrpl40* mutants (**H-H’**), and Chloramphenicol-treated (**F-F’**) larvae. Increased volume colocalizes with GFP signal in multiple brain regions in the ventricle-occupying midline.

Mitochondrial disruption leads to alterations in brain volume Individuals with 22q11.2DS exhibit alterations in brain size including a reduction in total brain volume and an increase in brain ventricular size [30, 31]. At the same time, metabolic genes, including those involved in mitochondrial function, have been previously implicated in regulating cortical growth and size [32]. Therefore, we examined whether disruption of mitochondrial functioning might cause behavioral phenotypes through an effect on brain volume. To determine if mitochondrial mutants and Cam-treated animals exhibited regional differences in brain volume we utilized deformation-based morphometry [33–35]. Briefly, larvae were raised to 6dpf, fixed, and stained for the brain structural marker total-ERK (tERK) [36]. Confocal stacks through the entire brain were then acquired on genotyped larvae and registered to a tERK reference brain [36]. Differences in deformation across the entire brain were computed for mutant/treated vs. wildtype/control larvae. Compared to controls, *prodha* mutant, *mrpl40* mutant, and Cam-treated (treated from 1-5dpf) larvae all displayed regional differences in brain volumes (Fig. 3A–C). Interestingly, differences appear similar across the two mutants and the mitochondrial translation inhibitor-treated larvae, with all three showing large reductions in the telencephalon and mesencephalon and smaller increases in the rhombencephalon (Fig. 3A–E). *prodha* mutants and Cam-treated larvae also show additional reductions in the diencephalon (Fig. 3D). We noted that reductions in brain volume were predominantly present in lateral brain regions, while increases tended to occur centrally with a distribution reminiscent of brain ventricles (Fig. 3A–C). To further assess this, we used the Zebrafish Brain Browser [37] to overlay our volumetric data onto a registered confocal stack of *Tg(gfap:GFP)*, which labels populations of ventricular neural stem and progenitor cells (NSPCs). This revealed significant colocalization between increased brain volume signal and *Tg(gfap:gfp)* across all three conditions in multiple brain areas, suggesting possible increases in brain ventricular size and/or alteration of NSPC populations which reside along the ventricles. Importantly, compared to wildtype larvae, we observed reduced apoptosis in the brains of both *mrpl40* and *prodha* mutants (Supplementary Fig. S2), suggesting that rather than apoptosis, deficits in neurogenesis may underlie the observed volumetric phenotypes.

### *mrpl40* and *prodha* play distinct roles in NSPC proliferation

**Fig. 4:**
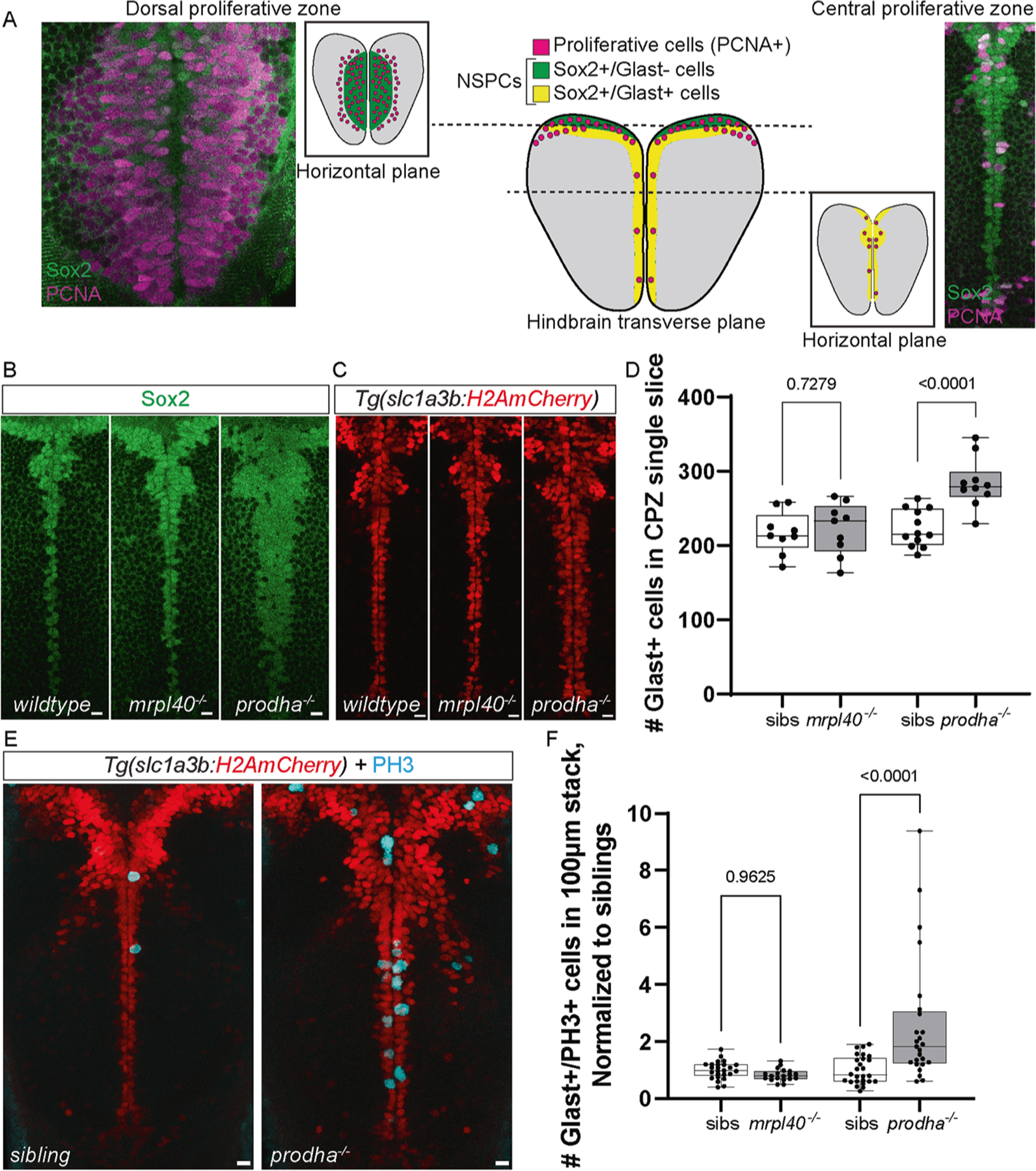
*prodha* mutant NSPCs in the central proliferative zone are expanded and hyperproliferative. **A** Diagram depicting NSPC populations in the zebrafish hindbrain at 5dpf. Transverse plane view shows highly proliferative NSPCs (Sox2+/Glast−) and committed progenitors/neuroblasts (Sox−/Glast−) located dorsally (Dorsal proliferative zone inset) and less proliferative NSPCs (Sox2+/Glast+) located centrally (Central proliferative zone inset). Insets are horizontal plane view. **B–D** Sox2 antibody staining (**B**) and *Tg(slc1a3b:H2AmCherry)* labeling (**C**) in central proliferative zone in wildtypes, *mrpl40* mutants, and *prodha* mutants. Wildtypes and *mrpl40* mutants display similar patterns whereas *prodha* mutants have an expanded Sox2+ and Glast+ domain. Images are single confocal slices. Scale bars 10 μm. Quantification shows increased number of Glast+ cells in *prodha* mutants (**D**). Unpaired two-way Student’s *t* test, *p*-values are displayed on the plots. *mrpl40* mutants (*n* = 9) and siblings (*n* = 9). *prodha* mutants (*n* = 10) and siblings (*n* = 12) from two biological replicates. E, F PH3 staining in central proliferative zone in siblings, *mrpl40* mutants, and *prodha* mutants. *prodha* mutants show an increased number of Glast+/PH3+ cells compared to siblings (F). Unpaired two-way Student’s *t* test, *p*-values displayed on the plots. Images are maximum projections from confocal stacks. Scale bars 10 μm. *mrpl40* mutants (*n* = 19) and siblings (*n* = 25). *prodha* mutants (*n* = 25) and siblings (*n* = 26) from four biological replicates.

**Fig. 5:**
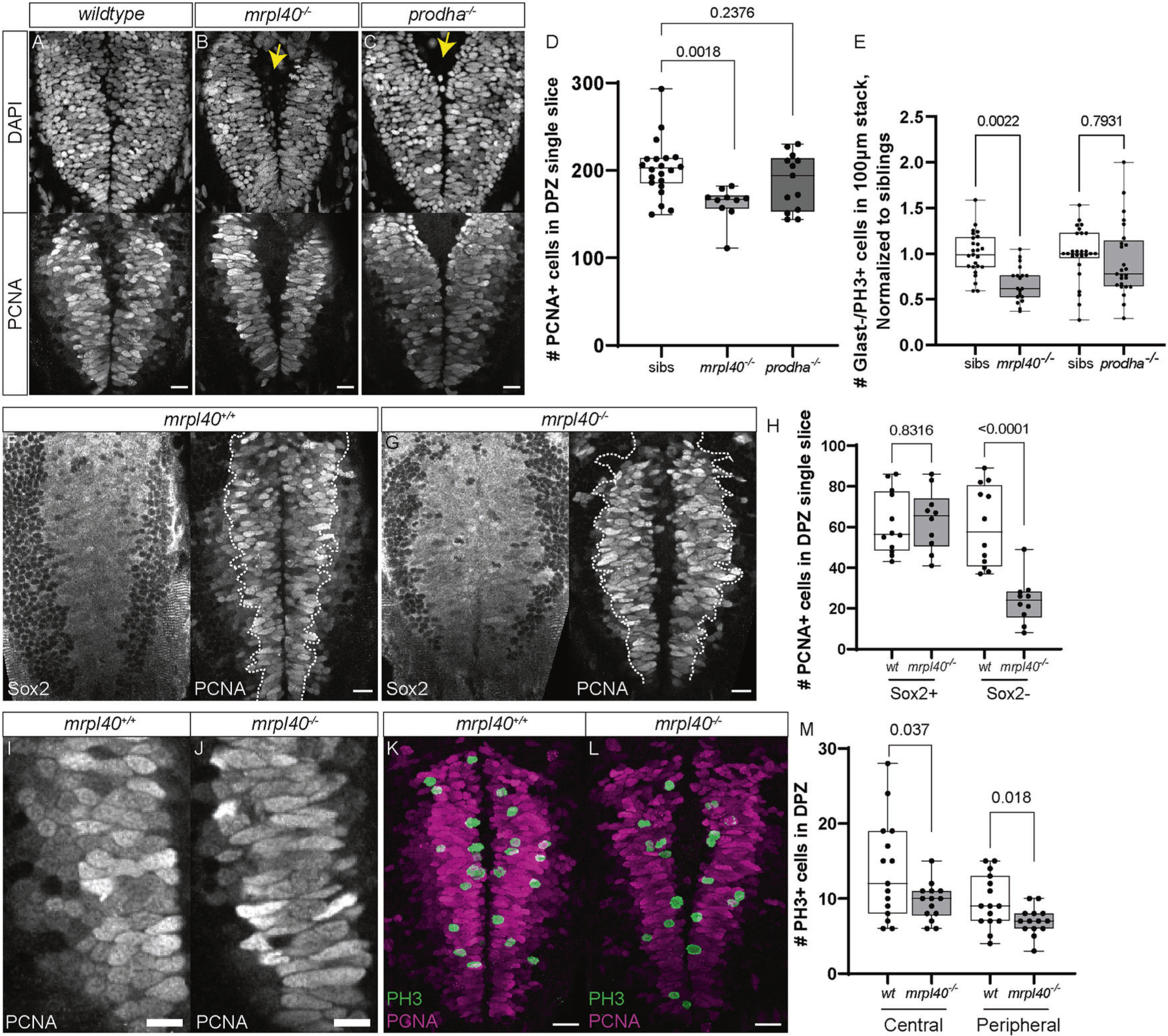
*mrpl40* mutants have fewer NSPCs and a reduction in NSPC proliferation in the dorsal proliferative zone. **A–D** PCNA and DAPI antibody staining in wildtypes (**A**), *mrpl40* mutants (**B**), and *prodha* mutants (**C**) in dorsal proliferative zone. *mrpl40* mutants have fewer PCNA+ cells than wildtypes. *prodha* mutants have similar numbers to wildtypes. Quantification in **D**. One-way ANOVA, *p*-values are displayed on the plots. Siblings (*n* = 21), mrpl40 mutants (*n* = 10), prodha mutants (*n* = 13) form two biological replicates. Both *mrpl40* and *prodha* mutants appear to have increased ventricle size (yellow arrows). Images are single confocal slices. Scale bars 15 μm. **E** PH3 staining in dorsal proliferative zone in siblings, *mrpl40* mutants, and *prodha* mutants.. *mrpl40* mutants show a decrease in the number of Glast−/PH3+ cells compared to siblings. Unpaired two-way Student’s *t* test, *p*-values displayed on the plots. *mrpl40* mutants (*n* = 19) and siblings (*n* = 25). *Prodha* mutants (*n* = 25) and siblings (*n* = 26) from four biological replicates. **F–H** Sox2/PCNA antibody staining in wildtypes (**E**) and *mrpl40* mutants (**F**) in dorsal proliferative zone. *mrpl40* mutants show a reduction in the number of Sox2−/PCNA+ cells but no difference in the number of Sox2+/PCNA+ cells (**G**). Quantification in **G**. Unpaired two-way Student’s *t* test, *p*-values displayed on the plot. *mrpl40* mutants (*n* = 10) and siblings (*n* = 12) from three biological replicates. Images are single confocal slices. Dotted line indicates boundary of Sox2+ cells. Scale bars 15 μm. **I–J** Proliferative cells in dorsal hindbrain of *mrpl40* mutants (**I**) display elongated nuclei compared to wildtypes (**H**). Images are single confocal slices. Scale bars 10 μm. **K–M** PH3/PCNA antibody staining in wildtypes (**J**) and *mrpl40* mutants (**K**) in dorsal proliferative zone. *mrpl40* mutants show a reduction in the number of PH3+ cells located both centrally along ventricle and more peripherally. Quantification in **L.** Unpaired two-way Student’s *t* tests, *p*-values displayed on the plot. *mrpl40* mutants (*n* = 14) and siblings (*n* = 15) from three biological replicates. Images are maximum projections from confocal stacks. Scale bars 20 μm.

Volumetric analysis of *mrpl40* and *prodha* mutants revealed significant reductions in brain volume and abnormalities surrounding brain ventricles, where NSPCs reside. Recent studies have implicated mitochondria as important regulators of neurogenesis [17], prompting us to consider the possibility that defective mitochondrial function in mutant animals may be linked to defects in NSPCs. To examine this possibility in more detail, we focused on the hindbrain, as both *mrpl40* and *prodha* mutants display significant volumetric changes in the hindbrain. Furthermore, the hindbrain contains neuronal populations that regulate ASR and visuomotor behaviors, which are disrupted in both mutants (Fig. 1). At 5dpf, hindbrain NSPCs marked by the multipotency marker Sox2 can be divided into two distinct populations based on expression of the glial marker *slc1a3b* (Glast) (Fig. 4A). First, Glast− NSPCs located in the dorsal hindbrain (dorsal proliferative zone, DPZ) are highly proliferative, with the majority expressing the cell proliferation marker Proliferating cell nuclear antigen (PCNA) (Figs. 4A and 5F). Second, Glast+ NSPCs lining the ventral, central ventricular surface (central proliferative zone, CPZ) are less proliferative (Fig. 4A) and express both *gfap* and *fat4* [38], indicating they likely represent radial glia-like cells. *mrpl40* and *prodha* are expressed in both of these cell populations, though *prodha* appears to be enriched in the CPZ (Supplementary Fig. S3), suggesting it may be particularly important in this cell population.

We first examined the CPZ in *mrpl40* and *prodha* mutants using wholemount immunofluorescence with an antibody for the NSPC marker Sox2 and a transgenic line that marks nuclei of Glast+ cells with mCherry (*Tg(slc1a3b:H2AmCherry*) [39]. While *mrpl40* mutants appeared indistinguishable from wildtypes, *prodha* mutants displayed an expanded CPZ, as shown by both Sox2 antibody staining and the *Tg(slc1a3b:H2AmCherry)* transgenic line (Fig. 4B, C). Quantification revealed an increased number of Glast+ cells in *prodha* mutants, but not *mrpl40* mutants (Fig. 4D). This expansion in *prodha* mutants is consistent with our volumetric measurements (Fig. 3A) that revealed increases in volume along the central hindbrain ventricle. To determine whether abnormal proliferation may be contributing to the CPZ expansion, we assessed expression of the mitosis marker phospho-histone H3 (PH3) in *Tg(slc1a3b:H2AmCherry)* animals. We observed a significant increase in the number of Glast+ mitotic cells in *prodha* mutants compared to siblings (Fig. 4E, F). Conversely, *mrpl40* mutants appeared indistinguishable from siblings (Fig. 4E, F). Together, this data indicates that *prodha* regulates proliferation and cell numbers of Glast+ NSPCs in the CPZ, while *mrpl40* appears to be largely dispensable for this process.

We next analyzed the DPZ in *mrpl40* and *prodha* mutants. PCNA staining revealed that while the number of proliferative cells was similar between wildtype and *prodha* mutants, there was a significant reduction in *mrpl40* mutants (Fig. 5A–D). Consistent with this, we also observed a significant reduction in the number of Glast−/PH3+ cells in *mrpl40*, but not *prodha*, mutants (Fig. 5E). In both mutants, however, DAPI nuclear staining revealed an enlarged ventricle (Fig. 5A–C), which may account, in part, for the increases in volume found in our volumetric experiments. To better understand the reduction of PCNA+ cell numbers in *mrpl40* mutants, and to determine which cell type(s) may be affected, we co-stained with Sox2 antibody. In wild-type animals, we observed Sox2+ cells located medially and expressing PCNA at high levels (PCNA^hi^). These PCNA^hi^/Sox2+ cells abutted weaker expressing PCNA cells (PCNA^lo^) laterally (Fig. 5F). In contrast to PCNA^hi^ cells which also expressed Sox2, PCNA^lo^ cells did not express Sox2 and their nuclei appeared smaller (Fig. 5F). This is consistent with the presence of at least two populations of proliferative cells in the DPZ, with PCNA^hi^/Sox2+ cells likely representing highly proliferative uncommitted/intermediate progenitors and PCNA^lo^/Sox2− cells representing committed progenitors or neuroblasts. In *mrpl40* mutants, we observed a specific reduction in the number of PCNA^lo^/Sox2− cells compared to wildtypes, while the number of PCNA^hi^/Sox2+ cells was unaffected (Fig. 5F–H). Interestingly, while the number of PCNA^hi^/Sox2+ cells was unaffected in *mrpl40* mutants, the PCNA^hi^/Sox2+ cells displayed abnormal morphology, appearing elongated compared to wildtypes (Fig. 5I, J). We therefore wondered whether a proliferative defect in PCNA^hi^/Sox2+ cells may be underlying the reduction in PCNA^lo^/Sox2− cell numbers. To test this idea, we analyzed the spatial distribution of PH3 expression within the DPZ. As PCNA^lo^/Sox2− cells are located laterally, we reasoned that PH3 expression in cells located centrally along the ventricle represent mitotic PCNA^hi^/Sox2+ cells, whereas PH3+ cells located more laterally are a mixture of PCNA^hi^/Sox2+ and PCNA^lo^/Sox2− cells. We counted PH3+ cells within the DPZ and grouped them based on whether the cells were located in the first cell layer adjacent to the ventricle or in more lateral regions. Consistent with the overall decrease in DPZ PCNA+ cells, in *mrpl40* mutants we observed a reduction in lateral PH3+ cells (Fig. 5K–M). Moreover, we also observed a reduction in central PH3+ cells, suggesting that reduced proliferation of PCNA^hi^/Sox2+ cells likely contributes to the observed reduction in PCNA^lo^/Sox2− cell numbers (Fig. 5K–M). Together, our results indicate that both *prodha* and *mrpl40* are required for NSPC proliferation, though they appear to regulate this process in distinct NSPC populations: *prodha* regulates proliferation of Glast+ cells in the CPZ, whereas *mrpl40* regulates proliferation of Glast− cells in the DPZ.

### *mrpl40* and *prodha* function independently from each other to regulate behavior and NSPC proliferation

*mrpl40* and *prodha* proteins are localized to mitochondria and mutants display similar behavioral and brain volumetric phenotypes, raising the possibility that they regulate behavior through a common genetic pathway. However, our single mutant NSPC analysis suggests these genes have independent functions in distinct cell populations. To test whether these genes function within a common pathway, we analyzed the behavior of double mutants for *mrpl40* and *prodha* across multiple behavioral metrics (Supplementary Fig. S4). If double mutants display the same phenotypic severity compared to single mutants, this indicates that the genes act within a common pathway. Conversely, if double mutants display a stronger phenotype, this is consistent with the genes acting in separate pathways. Analysis of *mrpl40* and *prodha* double mutants revealed more severe phenotypes than either single mutant alone in both the VMR and DF assays (Supplementary Fig. S4A–C). For example, double mutants had lower average speed in the dark phase of the VMR assay (Supplementary Fig. S4B) and lower fraction of O-bends in the DF assay (Supplementary Fig. S4C) than either single mutant alone. In the ASR assay, where *mrpl40* and *prodha* single mutants have opposite phenotypes (*mrpl40* mutants have lower ASR sensitivity and *prodha* mutants have higher ASR sensitivity), double mutants displayed an intermediate phenotype and were not significantly different from siblings (Supplementary Fig. S4D). Notably, in any of the assays examined, transheterozygous animals were indistinguishable from wild-type or single heterozygote animals (Supplementary Fig. S4A–D). Together, these results indicate that while single mutants of *mrpl40* and *prodha* have similar behavioral and brain morphology phenotypes, they function in genetically distinct pathways.

**Fig. 6:**
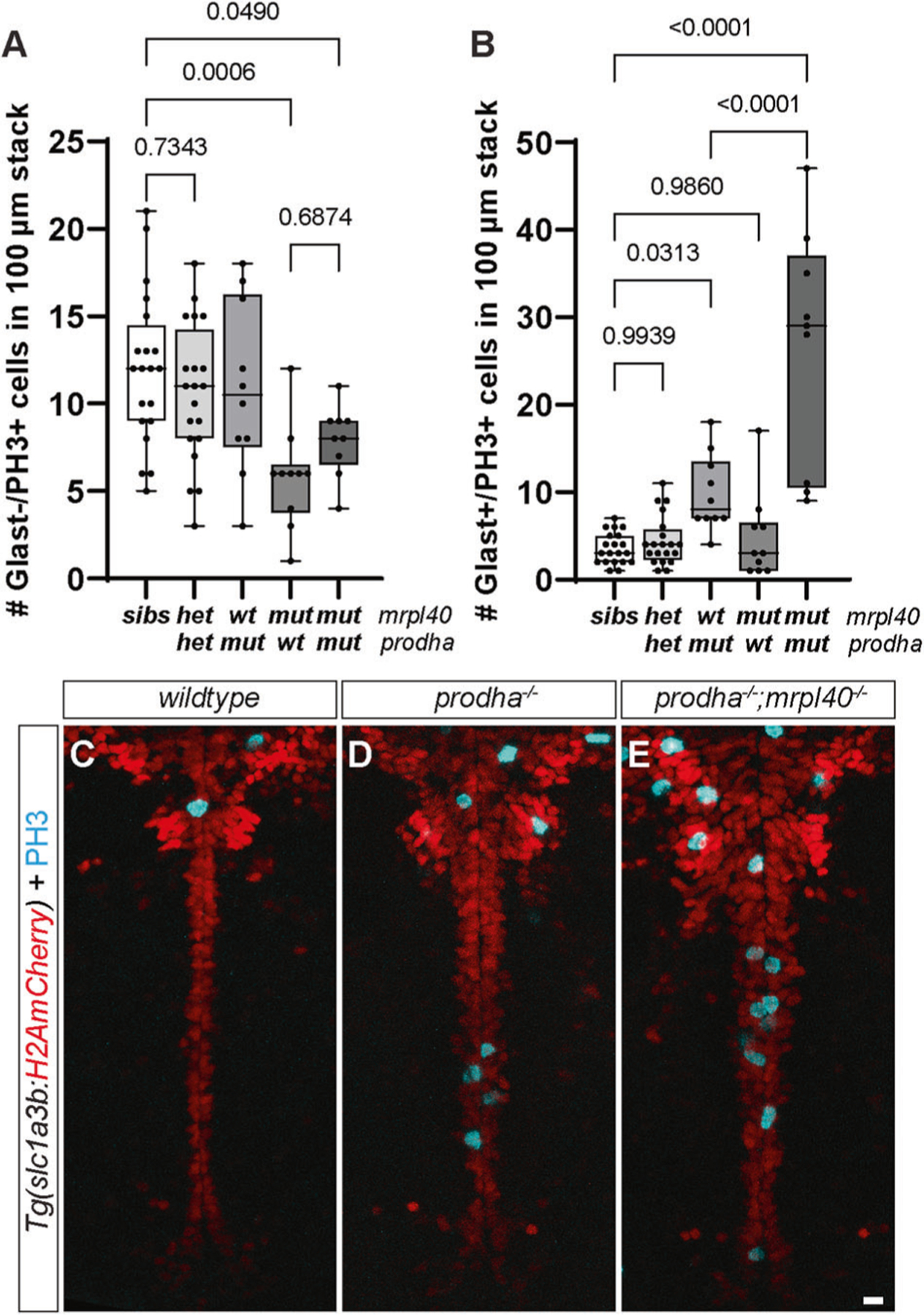
*mrpl40* and *prodha* function independently from each other to regulate NSPC proliferation. Number of Glast−/PH3+ (**A**) and Glast+/PH3+ (**B**) cells in *mrpl40/prodha* transheterozygotes and double mutants compared to single mutants. Double mutants have both *prodha* and *mrpl40* single mutant phenotypes. Transheterozygotes display no phenotypes. Quantification one-way ANOVA, *p*-values are displayed on the plots. siblings (*n* = 20), transheterozygotes (*n* = 20), *mrpl40* mutants (*n* = 10), *prodha* mutants (*n* = 10) and *mrpl40;prodha* double mutants (*n* = 9) from four biological replicates. PH3 staining in *Tg(slc1a3b:H2AmCherry)* line in wildtype (**C**), single *prodha* mutants (**D**), and double mutants (**E**). Double mutant has more severe hyperproliferative phenotype, revealing role for *mrpl40* in Glast+ NSPCs.

These results are consistent with our NSPC findings where we observe roles for *mrpl40* and *prodha* in distinct NSPC populations. To confirm these distinct phenotypes in double mutants at the level of NSPCs, we assessed PH3 expression in Glast+ and Glast− cells as we did previously for single mutants. Consistent with roles in two distinct pathways, we observed that double mutants had both NSPC phenotypes: the lower number of Glast−/PH3+ cells of *mrpl40* mutants (Fig. 6A) and the higher number of Glast+/PH3+ cells of *prodha* mutants (Fig. 6B). Unexpectedly, however, we found that double mutants had a striking increase in the number of Glast+/PH3+ cells even compared to *prodha* mutants (Fig. 6B–E), indicating that *mrpl40* also regulates Glast+ cell proliferation. Notably, we observed no difference in the number of Glast+ or Glast− proliferative cells in transheterozygotes. These results, together with our behavioral analysis, support the notion that during development *prodha* and *mrpl40* regulate partially overlapping brain volumes and behaviors by regulating proliferation of distinct NSPC populations. Taken together, our data reveal a previously unappreciated link between 22q11.2DS-relevant behavioral phenotypes, mitochondrial function, and neurogenesis.

## DISCUSSION

22q11.2DS predisposes individuals to multiple NDDs and represents one of the greatest risk factors for schizophrenia, yet it has been difficult to define which genes within the region contribute to brain and behavioral phenotypes. Part of this difficulty is likely due to the idea that rather than single genes acting alone to impart disease risk, multiple genes within the deleted region act together through shared biological pathways/functions to drive pathology. However, the identity of these shared pathways and the relevant genes has remained unclear. Here, we identify previously unappreciated roles for the *mrpl40* and *prodha* genes in NSPC proliferation and provide compelling evidence that both genes function in genetically separable pathways to regulate mitochondrial-supported NSPC proliferation. Furthermore, while our results support previous studies implicating mitochondrial function in 22q11.2DS pathology, they indicate that defects in NPSCs precede post-mitotic defects. Finally, our results provide a first functional link between mitochondrial gene dysfunction, brain volumetric changes, and 22q11.2DS-relevant behavioral phenotypes. Thus, our results strongly support the notion that mitochondrial dysfunction plays an early and central role in the etiology of neurodevelopmental disorders associated with 22q11.2DS.

Previous studies have suggested that mitochondrial dysfunction is involved in 22q11.2DS pathogenesis. Indeed, at least six of the genes in the deleted region have been shown to localize to mitochondria [10], with several displaying behavioral phenotypes in mouse knockouts [14, 15, 40, 41]. Further, iPSC-derived neurons from patients with the deletion display reduced levels of electron transport chain components and ATP [11, 12]. Despite evidence that suggests NSPC proliferation may be defective in 22q11.2DS [3, 42, 43], studies to date have focused on mitochondrial roles in post-mitotic neurons. Our data indicate that two evolutionarily conserved mitochondrial genes within the 22q11.2 deleted region are required for zebrafish NSPC function, implicating NSPC mitochondrial dysfunction as a potential mechanism in 22q11.2DS pathology. We hypothesize that mitochondrial dysfunction resulting from cumulative effects of deleterious mutations in multiple genes in the region results in NSPC deficits. Indeed, our double mutant analysis indicates a partially redundant role for *mrpl40* and *prodha* in regulating radial glia-like proliferation. Furthermore, a recent study demonstrated that *mrpl40* and another mitochondrial gene in the deleted region, *slc25a1*, biochemically interact to maintain mitochondrial ribosomal integrity [44]. Our hypothesis is also supported by an increasing recognition of mitochondria as key regulators of NSPC proliferation and neurogenesis [17, 45] and recent data suggesting that mitochondrial dysfunction may serve as a general pathological mechanism underlying multiple NDDs [25, 46]. Therefore, understanding the roles that mitochondria serve in NSPCs is crucial to furthering our understanding of 22q11.2DS and NDDs in general.

Mitochondrial ribosomal protein L40 (*mrpl40*) is a component of the mitochondrial ribosome that is required for translation of mitochondrially encoded genes, many of which are involved in oxidative phosphorylation (OxPhos). Previously, *mrpl40* has only been examined in the context of post-mitotic neuron function. Heterozygous loss of *mrpl40* in cultured forebrain-like excitatory neurons leads to reductions in OxPhos complexes and ATP levels that phenocopy deficits observed in 22q11.2DS patient-derived neurons [11]. Heterozygous *mrpl40* mouse knockouts display deficits in working memory that have been attributed to abnormal intracellular calcium buffering [47]. Knockdown of *mrpl40* in *Drosophila* leads to abnormal larval neuromuscular junction synapse development and function and sleep phenotypes [44]. Here we show a previously unrecognized role for *mrpl40* in regulating brain size and NSPC proliferation. Due to its role in OxPhos gene translation, we hypothesize that mitochondrial OxPhos genes are required for NSPC proliferation and maintenance of brain volume. This is supported by our mitochondrial translation inhibition experiments that reveal behavioral and brain volume changes similar to those we observe in *mrpl40* mutants. This is also consistent with data from *Drosophila* [48] and mouse [49, 50] studies that found that disrupting OxPhos reduces NSPC proliferation. Our results provide a more granular view in that different populations of NSPCs exhibit differential sensitivity to the loss of *mrpl40* function. Specifically, *mrpl40* mutants display reduced NSPC proliferation in the DPZ of the hindbrain, while Glast+, radial glia-like cells in the CPZ appear unaffected. One possible explanation for this is that NSPC populations have different reliance on mitochondria. Indeed, multipotent neural stem cells are mainly glycolytic and shift to relying on mitochondria as intermediate progenitors and during commitment [51, 52]. Therefore, it is possible that radial glia-like CPZ NSPCs are more reliant on glycolysis whereas DPZ NSPCs require mitochondria. Another possibility may relate to differences in proliferation between these populations. Specifically, the more highly proliferative nature of DPZ NSPCs may mean that they are more reliant on mitochondria. In support of such a mechanism is the observation that OxPhos disruption alters cell cycle dynamics [48, 53], so it is possible that highly proliferative cells may be more vulnerable to mitochondrial dysfunction. Importantly, our results also indicate that in the context of *prodha* loss, *mrpl40* functions to repress CPZ NSPC proliferation. This surprising finding that *mrpl40* can function in opposing directions depending on NSPC cell type is intriguing and warrants further investigation. Understanding how the loss of *mrpl40* function impacts downstream events, and determining the specificity of the NSPC proliferative phenotypes are important questions that remain unanswered.

Proline Dehydrogenase (Prodh) is localized to mitochondria and is responsible for the first step of L-Proline metabolism. Humans with homozygous mutations in *PRODH* display elevated levels of L-Proline as well as multiple NDDs including developmental delay, ID, ASD, and SZ [54]. To date, studies of *PRODH*’s role in brain function and behavior have mainly focused on the role of hyperprolinemia in post-natal, post-mitotic neurons [13, 14]. Our results indicate that *prodha* plays an important role in NSPC development, in line with observed NDD clinical phenotypes. Specifically, we find that *prodha* mutants display a reduction in brain size, an expanded CPZ, and hyperproliferative Glast+ NSPCs. Although its role in NSPC development has not been previously examined, *PRODH* has been extensively investigated in other highly proliferative cells, such as cancer cells where it can act as either a tumor suppressor or oncogene depending on context [55]. Although the NSPC phenotypes we uncovered in *prodha* mutants provide insights into the role of *prodha* in NSPCs, there are several potential mechanisms by which *prodha* might act. For example, L-Proline oxidation via *prodha* leads to reducing equivalents of NADH/FADH_2_ that can feed OxPhos to serve NSPC energetic needs. Alternatively, *prodha* may regulate NSPCs through reactive oxygen species (ROS). Prodh is known to promote ROS formation in several contexts [56–58] and low levels of exogenous ROS have been shown to promote proliferation of NSPCs [59] and other stem cells [60]. L-Proline’s metabolism also leads to glutamate, which can contribute to the TCA cycle and play critical roles for forming other cellular substates important for highly proliferative cells. Furthermore, the TCA cycle has recently been identified as a key hub of cell fate decisions in embryonic stem cells [61], so it is possible that TCA substrates may perform more instructive roles during neurogenesis.

In addition to the five genes that that we identified as having roles in behavior, our unbiased approach also identified six mutants with gross morphological phenotypes: *ess2*, *ufd1l*, *cdc45*, *tbx1*, *med15*, and combination deletion of *pi4kaa/slc25a1b* (Supplemental Table 2). 22q11.2DS is a multisystem syndrome and individuals can display abnormalities beyond brain and behavioral phenotypes, including cardiovascular and craniofacial abnormalities. Consistent with this, four of these mutants displayed heart edema (*ess2*, *tbx1*, *med15*, *pi4kaa/slc25a1b*) and two had gross jaw abnormalities (*tbx1*, *med15*). It is important to note that while we analyzed behavior of heterozygotes for these genes and found no phenotypes, this does not exclude important roles for these genes in brain development or behavior. Indeed, four of these homozygous mutants displayed gross eye or brain phenotypes (*ufd1l*, *cdc45*, *med15*, *pi4kaa/slc25a1b*) and warrant further investigation for their roles in neurodevelopment.

One limitation to this study that should be considered is that we have focused on homozygous mutants whereas patients with 22q11.2DS are heterozygous for genes in the deleted region. However, it is not unusual for genes associated with disease in humans in the heterozygous state to require homozygosity in zebrafish larvae in order for phenotypes to be observed [62, 63]. One possible explanation for this observation is that a large number of gene products that are deposited in the zebrafish egg function during early development and persist for a period of time as development progresses. Indeed, there is RNA sequencing evidence that all of the five genes identified in our study are expressed in the ovary and early embryo prior to zygotic gene activation [28]. As such, zygotic homozygous mutants that retain maternal gene products may be somewhat similar to the human heterozygous state. While this distinction between the zebrafish and human conditions should be considered when interpreting our results, our data nonetheless indicate that all of the genes we have identified do contribute to establishing or maintaining behavior in the zebrafish model.

Together, our data present NSPC proliferation defects as a potential core component of 22q11.2DS pathology. Our work complements previous studies that have shown that two other genes in the deleted region, *dgcr8* [64, 65] and *ranbp* [66], also affect NSPC proliferation. It remains to be determined to what extent NSPC proliferation deficits lead to behavioral outcomes and through which mechanisms. To fully appreciate how mitochondrial dysfunction caused by loss of *mrpl40* and *prodha* may lead to behavioral phenotypes will require careful examination of progenitor and post-mitotic cell diversity.

## MATERIALS AND METHODS

### Experimental model details

Experiments were conducted on 5–6 dpf larval zebrafish (Danio rerio, TLF strain) raised in E3 medium at 29 °C on a 14:10 h light cycle. At this developmental stage the sex of the organism is not yet determined. Breeding adult zebrafish were maintained at 28 °C on a 14:10 h light cycle. Sample size for each experiment was estimated based to ensure ability to observe effect while minimizing animals used. Animals were not randomized. Mutant alleles were generated using CRISPR-Cas9 mutagenesis. For single gene mutants, two gRNAs flanking a known functional domain or highly conserved region were designed using ChopChop v2 (https://chopchop.cbu.uib.no/). Alt-R CRISPR-Cas9 crRNAs (IDT) were annealed with tracrRNA (IDT), complexed with Cas9 protein (PNA Bio, CP02), and injected into 1-cell stage embryos. F_0_ injected larvae were raised and outcrossed to identify and establish heterozygous carrier lines. For combination gene mutants, four gRNAs were used: two gRNAs targeting the gene flanking one end of the deletion were injected together with two gRNAs targeting the gene flanking the other end in order to delete the intervening region. Mutant lines were identified and subsequently genotyped by PCR, using primers flanking the gRNA target sites. Allele sequences were obtained by Sanger-sequencing of PCR products. For a subset of alleles, genotyping was also performed with the KASP method with proprietary primer sequences (LGC Genomics). This method was validated using the PCR method. Transgenic lines used in this study were as follows: *Tg(slc1a3b:myrGFP-P2A-H2AmCherry)*^*vo80Tg*^ provided by Kelly Monk, Oregon Health & Science University [39]; *Tg(sox2-2A-sfGFP)*^*stl84Tg*^ provided by Benjamin Martin, Stonybrook University [67]; *Tg(NBT:DsRed)*^*zf148Tg*^ [68]. All animal protocols were approved by the University of Pennsylvania Institutional Animal Care and Use Committee (IACUC).

### Behavior recording

Behavior experiments were performed on 6dpf larvae. For each behavioral run, approximately 50 larvae were used for each mutant allele. Larvae were arrayed in 100-well plates fabricated from 2 mm thick clear acrylic sheets (McMaster-Carr). Wells were 9 mm in diameter and 2 mm in depth and arrayed 10 × 10. The plate was illuminated from below with IR-850nm LED arrays (IR100, CMVision) which were diffused using a white acrylic sheet. Images were recorded from above with 1024 × 1024 resolution using a Photron UX50 camera, Quantaray 50 mm 1: 2.8D Macro for Nikon AF lens, and IR long-pass filter (LP800-55, Midwest optical). To provide acoustic vibrational stimuli (2 ms duration, 1000 Hz waveforms), the plate was attached via an aluminum rod to a vibrational exciter (4810; Brüel and Kjaer, Norcross, GA). White light (MCWHL2, Thorlabs) was provided from above (700 mW, 6500 K). Video recording, white light LED, and vibrational stimuli were triggered by a NI PCI-6221 M Series DAQ [24].

The full behavioral paradigm consisted of the following described blocks in the stated order. VMR: Lights OFF (10 min, unrecorded) > Lights ON (8 min, 20fps) > Lights OFF (8 min, 20fps) > Video saved (~12 min). LF: 15 stimuli of white light of 500 ms duration with 30 s interstimulus interval (ISI). Recording 500 ms post-stimulus at 500fps (8 min) > Video saved (~2 min). DF: Lights ON (5 min, unrecorded) > 14 stimuli of darkness of 1 s duration with 30 s ISI (recorded) > 14 stimuli of darkness of 1 s duration with 10 s ISI (unrecorded) > 14 stimuli of darkness of 1 s duration with 10 s ISI (recorded) > 14 stimuli of darkness of 1 s duration with 10 s ISI (unrecorded) > 14 stimuli of darkness of 1 s duration with 10 s ISI (recorded). Recording 1 s post-stimulus at 500fps (~23 min total) > Video saved (~12 min). ASR: 10 stimuli of low intensity with 20 s ISI > 10 stimuli of medium intensity with 20 s ISI > 10 repetitions of the following with 20 s ISI PP1-PPI2-PPI3-PPI4-High Intensity (where PPI1: low intensity prepulse, 50 ms prior high intensity, PPI2: low intensity prepulse, 300 ms prior high intensity, PPI3: medium intensity prepulse, 50 ms prior high intensity, PPI4: medium intensity prepulse, 300 ms prior high intensity, High Intensity: no prepulse) > 30 stimuli of high intensity with 1ISI. Recording 200 ms post-stimulus at 500fps (~24 min total) > Video saved (~6 min). The behavioral paradigm took place over an approximately two-hour period during the daytime hours from 8AM to 4PM. Following behavioral experiments, larvae were individually genotyped as described above.

### Larval tracking

Offline tracking of recorded videos was performed with Python. Each image was background subtracted, Gaussian blurred, thresholded, and the centroid identified to identify larvae. For LF, DF and ASR assays five points along the tail were also identified as previously described [69]. The orientation of the larvae was determined as the vector between the first point along the body and the centroid and the curvature was defined as the sum of the angles between all of the points along the larva.

### Behavioral metrics

A total of 94 metrics were computed for each behavioral run and they are described below.

#### VMR (60).

Number of bouts, Total distance traveled (pix), Total time moved (ms), Average speed (pix/ms), Average distance from center of the well (pix), Fraction of time spent in out rim of well, Average distance/bout (pix), average displacement/bout (pix), Average time/bout (ms), Average speed/bout (pix/ms). A bout was defined as the displacement of the centroid crossing a certain threshold (0.7 pixels) for at least two consecutive frames. For a bout to end, two consecutive frames had to have a displacement below this threshold. Centroid movements were only considered if they occurred during periods defined as bouts. Behavioral metrics were considered for the first and last minutes of the Light ON and Light OFF segments and for the entirely of each of the Light ON and Light OFF segments for a total of six bins for each metric, and a total of 60 metrics for the VMR assay

#### LF (9).

Frequency of reacting, frequency of no movement, Average latency to movement (ms), Average maximum bend angle (rad), Average maximum angular velocity (rad/frame), Average change in orientation (rad), Average displacement (pix), Average distance (pix), Average duration (ms). Larvae were classified as reacting if their maximum curvature was > 0.5 rad and latency of movement was not too early (>15 ms).

#### DF (11).

In addition to the metrics for LF, Frequency of O-bend response and Habituation of the O-bend response were calculated. Larvae were classified as performing an O-bend if their maximum curvature was >1.75 rad or reacting if it was >0.5 rad but less than 1.75 rad as long as latency of movement was not too early (>15 ms). O-bend habituation was calculated as (1 − [Obend frequency for last 14 stimuli]) ÷ [Obend frequency for first 14 stimuli].

#### ASR (14).

In addition to the metrics for LF, Frequency of SLC response, Frequency of LLC response, Sensitivity Index of the ASR (AU), Pre-pulse inhibition of the ASR, and Habituation of the ASR were calculated. Larvae were classified as performing an SLC if their maximum curvature was >0.8 rad and the latency was <20 ms, performing an LLC is their maximum curvature was >0.8 rad and the latency was >20 ms, or reacting if it was >0.5 rad but less than 0.8 rad. ASR habituation was calculated as (1-[SLC frequency for last 10 High intensity stimuli with 1 s ISI]) ÷ [SLC frequency for 10 High intensity stimuli with 20 s ISI]. ASR PPI was calculated as (1-[SLC frequency for 10 PPI4 runs]) ÷ [SLC frequency for 10 High intensity stimuli with 20 s ISI]. ASR Sensitivity index was calculated by measuring the area under the curve of SLC frequency versus stimulus intensity.

### Chloramphenicol treatment

Embryos from *mrpl40* heterozygous in crosses were raised in E3 medium, dechorionated, and treated with either Chloramphenicol (C0328, Sigma) dissolved in E3 or E3 medium alone starting at 1dpf. Media was replaced daily up until 6dpf when behavioral experiments were performed. Larvae were genotyped following behavioral analysis. Behavior experiments were performed using serial dilutions of Chloramphenicol to show dose-response (375–750 μM), whereas dose used for brain volumetric analysis was 562 μM.

### Deformation-based morphometry

Deformation-based morphometry was performed as described previously [35]. Briefly, whole-brain confocal stacks were registered to the previously reported 6-dpf *nacre* mutant (*mifta*^−*/*−^*)* larvae stained with anti-total ERK using CMTK (http://www.nitrc.org/projects/cmtk/) via the Fiji-CMTK-registration-runner-GUI (https://github.com/sandorbx/Legacy-Fiji-CMTK-registration-runner-GUI) with the following parameters: Threads 8, Initial exploration step 52, Coarsest resampling 8, Refine grid 3, Grid size 80, Accuracy 1. The jacobian was calculated using reformatx in CMTK, followed by smoothing the images with a custom Fiji script PrepareJacobianStacksForMAPMapping_cluster.m as previously described [35] (github repository: sthyme/ZFSchizophrenia/cluster_pErk_imageprocessing/). To compare volumetric stacks with *gfap* expression, the *Tg(gfap:gfp)* reference stack was obtained from the Zebrafish Brain Browser (http://vis.arc.vt.edu/projects/zbb/) and merged with volumetric stacks using Fiji.

### Immunohistochemistry

Larvae were raised to 5–6dpf at which point they were fixed in 4% paraformaldehyde in PBS. Larvae were stained as previously described [36]. Briefly, larvae were washed with PBS + 0.25% Triton X-100 (PBT) and pigment was removed using a 1.5% H_2_O_2_/0.5% KOH solution at room temperature (RT) for 15 mins followed by further PBT washes. For experiments with transgenic lines, embryos were raised in 0.003% PTU to remove pigment and the H_2_O_2_/KOH bleaching step was omitted to ensure fluorophores persisted. Antigen recovery was performed with 150 mM Tris-HCl, pH 9 for 5 min at RT followed by 15 min at 70 °C, followed by PBT washes. Permeabilization was performed with 0.05% Trypsin-EDTA for 5 min on ice followed by PBT washes. Larvae were then blocked with PBT + 1%bovine serum albumin + 2% normal goat serum (NGS) + 1% DMSO for 1 h at RT followed by overnight incubation at 4 °C with primary antibodies and DAPI in blocking solution without NGS. The following day PBT washes were performed followed by overnight incubation at 4 °C with secondary in blocking solution without NGS. Following further PBT washes, larval tails were removed with a razor blade and used for genotyping. Following genotyping, larvae were mounted in 1.5% low melting-point agarose in PBS dorsal side down in a glass-bottomed dish and imaged with an inverted confocal microscope (Zeiss LSM880 or LSM980) using either a 20X air objective for whole brain analysis, or a 40X water objective for hindbrain analysis. Imaging was not performed blinded to genotype. Primary antibody concentrations used were: Mouse Anti-PCNA Antibody (Sigma, MAB424, 1:500), Rabbit Anti-active Caspase-3 (BD Bioscience, 559565, 1:500), Rabbit Anti-Sox2 (Abcam, ab97959, 1:200), Rabbit Anti-phospho-Histone H3 (Sigma, 06-570, 1:500), Mouse Anti-total-ERK (Cell Signaling, 4696, 1:500). Alexa Fluor secondary antibodies were used at 1:500 dilution (Invitrogen).

### HCR fluorescent in situ hybridization

Endogenous *mrpl40* and *prodha* transcripts were detected using HCR fluorescent in situ hybridization (Molecular Instruments, Los Angeles, CA, USA). HCR buffers, probes, and hairpins were purchased from Molecular Instruments. *Tg(sox2-2A-sfGFP);Tg(NBT:DsRed)*-positive larvae were fixed at 5dpf with 4% paraformaldehyde in Dulbecco’s phosphate-buffered saline overnight at 4 °C and staining was performed as previously described [38].

### Quantification and statistical analysis

#### Behavioral quantification.

Behaviors were quantified, analyzed, and visualized with Python. Approximately 50 larvae from a heterozygous incross for each mutant allele were run through the behavior paradigm. Following genotyping, mutant larvae were compared to sibling larvae across all 94 behavioral metrics with two-tailed, unpaired Student’s *t* tests. If any behavioral metric had *p* < 0.05, an additional independent experiment was performed (expect for *cdc45* where only a single experiment was performed for technical reasons). In this independent experiment, *p* values were compared with the first experiment to identify if any behavioral metrics were in common. This was the case for 13 mutant lines and they had varying numbers of metrics in common ranging from 1 to 53. Those mutants that only had differences in metrics in the VMR assay and which only occurred within a single 1-min bin of time and in no others during the 16 min assay (*slc25a1b* (1 metric) and *scarf2* (4 metrics)) were felt to less likely represent true differences and were not considered further. To account for multiple comparisons, the data for both experiments was then pooled and subjected to further statistical stringency using a Bonferroni correction of *p* = 0.05/94. Prior to pooling, to account for run-to-run variation in behavior, behavioral metrics were first normalized within their experiment by dividing all values by the average sibling value. Normalized values were then pooled and statistics performed. Nine mutants had behavioral metrics that survived correction. One of the nine mutants (*dgcr2*), was significant only for habituation of the SLC response in the ASR assay. However, due to technical limitations and loss of the line, the ASR assay was only performed once. Since habituation of the SLC was only significant for the last 10 stimuli of the habituation paradigm and not the first 10 or the middle 10 stimuli, this was deemed less likely to be a true difference. The eight remaining mutants had a range of 5–47 behavioral metrics below the corrected *p* value. These are the data represented by the heatmaps shown in Fig. 2G and Supplementary Fig. S1.

### Deformation-based morphometry

Deformation-based morphometry was analyzed as described previously [35]. The Mann–Whitney U statistic Z score is calculated for each voxel, comparing between the mutant/treated groups and control groups. The significance threshold was set based on a false discovery rate (FDR) where 0.005% of control pixels would be called as significant. Following this, the extent to which volumetric changes overlapped within different Z-Brain atlas defined regions was performed by processing the volumetric stacks with the previously reported “ZBrainAnalysisOfMAPMaps.m” Matlab function [36].

### Immunohistochemistry quantification

All image visualization and processing was performed with Fiji. *Activated Caspase 3:* Positive cells were manually counted by manually progressing through stacks. The forebrain, tectum, and hindbrain were defined by anatomical landmarks visualized with DAPI co-staining. *Glast*+ *cells in central PZ:* For Fig. 4C, D, a single confocal slice at a comparable anatomical location ~50μm below dorsal surface of hindbrain was used for analysis. *H2AmCherry* signal was gaussian blurred, maximum filtered with radius of 5pixels, unsharp masked with radius 5 pixels and mask weight 0.6, thresholded, and then watershed function used. Nuclei were subsequently counted with the Analyze Particles function. *PH3 in central and dorsal PZ:* For Figs. 4E, F and 5E, PH3+ cells were manually counted as Glast+ or Glast− based on transgene expression by manually progressing through a 100 μm thick confocal stack through the hindbrain. DAPI/PCNA co-staining allowed visualization of the central ventricle. Number of PH3+ cells was normalized by dividing the observed number of PH3+ cells by the average number of PH3+ cells in siblings on that experimental day. For Fig. 5K–M, Positive cells were manually counted by manually progressing through stacks. DAPI co-staining allowed for distinction of those that were within the first cell layer along the ventricles vs. those that were located more peripherally. *Sox2/PCNA in dorsal PZ:* For Fig. 5A–D, a single confocal slice at a comparable anatomical location ~10 μm below dorsal surface of hindbrain was used for analysis. DAPI signal was gaussian blurred, maximum filtered with radius of 2pixels, unsharp masked with radius 5 pixels and mask weight 0.9, thresholded, and then watershed function used. PCNA signal was then thresholded and used to mask DAPI image, to leave only PCNA+ nuclei which were subsequently counted with the Analyze Particles function. For Fig. 5F–H, Sox2+/PCNA+ and Sox2−/PCNA + cells were manually counted in an ROI encapsulating a 93µmx122µm segment of one of the lateral hindbrain PCNA+ segments in a single z-slice ~10 µm below the dorsal most aspect of the hindbrain. *Hindbrain PH3:* One-way ANOVA, Chi-square, or two-tailed, unpaired Student’s *t* tests were used for immunohistochemistry statistics as indicated in Figure legends.

## Supplementary Material

Supplemental File 4

Supplemental File 3

Supplemental File 2

Supplemental File 1

Supplemental Table 7

Supplemental Table 6

Supplemental Table 5

Supplemental Table 4

Supplemental Table 3

Supplemental Table 2

Supplemental Table 1

Supplemental Figure 4

Supplemental Figure 3

Supplemental Figure 2

Supplemental Figure 1

Supplemental Table and File titles and legends
