# Supplementary material for: Mitochondrial proteins encoded by the 22q11.2 neurodevelopmental locus regulate neural stem and progenitor cell proliferation
Source: Mol Psychiatry. Author manuscript; Available in PMC 2024 Feb 16. (PMC10730408; doi:10.1038/s41380-023-02272-z)
Supplement: Supplemental Table 3 [file NIHMS1950658-supplement-Supplemental_Table_3.docx]

**Supplemental Table 3.** Incompletely penetrant swim bladder inflation phenotypes observed. Related to Figure 1.

| **Mutant line** | **Details from heterozygous in-cross** |
| --- | --- |
| *dgcr8* | Cross1: n=13/19 mutants without swim bladder. Cross2: n=10/14 mutants without swim bladder. Cross3: n=11/17 mutants without swim bladder. Cross4: n=9/14 mutants without swim bladder. |
| *C5del2* | Cross1: n=7/16 mutants without swim bladder. |
| *snap29* | Cross1: n=0/7 mutants without swim bladder. Cross2: n=9/11 mutants without swim bladder. Cross3: n=12/12 mutants without swim bladder. Cross4: n=9/11 mutants without swim bladder. |
| *C8del1* | Cross1: n=0/6 mutants without swim bladder. Cross2: n=0/8 mutants without swim bladder. |
