# Supplementary material for: Mitochondrial proteins encoded by the 22q11.2 neurodevelopmental locus regulate neural stem and progenitor cell proliferation
Source: Mol Psychiatry. Author manuscript; Available in PMC 2024 Feb 16. (PMC10730408; doi:10.1038/s41380-023-02272-z)
Supplement: Supplemental Table 2 [file NIHMS1950658-supplement-Supplemental_Table_2.docx]

**Supplemental Table 2.** Morphological phenotypes observed in homozygous mutants. Related to Figure 1.

| **Mutant line** | **Details from heterozygous in-cross** | **Phenotype description of homozygotes** |
| --- | --- | --- |
| *ess2* | Cross1: n=27/27 phenotypically normal larvae at 5dpf are het/wt.  Cross2: n=48/48 phenotypically normal larvae at 5dpf are het/wt. | 3dpf: heart edema, small and curved tail; n=4/4 with phenotype are mutant |
| *ufd1l* | Cross1: n=48/48 phenotypically normal larvae at 5dpf are het/wt.  Cross2: n=48/48 phenotypically normal larvae at 5dpf are het/wt. | 5dpf: curved back, dark brain; n =4/4 with phenotype are mutant |
| *cdc45* | Cross1: n=43/43 phenotypically normal larvae at 5dpf are het/wt.  Cross2: n=27/41 phenotypically normal larvae at 5dpf are het/wt. | 2dpf: curved tail, small eyes, dark brain; Incompletely penetrant; n =12/12 with phenotype are mutant |
| *tbx1* | Cross1: n=41/41 phenotypically normal larvae at 5dpf are het/wt. | 3dpf: heart edema, jaw abnormalities; did not genotype phenotypic larvae as phenotype has been previously reported for *tbx1* mutants |
| *med15* | Cross1: n=35/35 phenotypically normal larvae at 5dpf are het/wt.  Cross2: n=40/40 phenotypically normal larvae at 5dpf are het/wt. | 5dpf: small eyes, heart edema, jaw abnormalities; n =16/16 with phenotype are mutant |
| *C10del2* | Cross1: n=33/33 phenotypically normal larvae at 5dpf are het/wt.  Cross2: n=38/38 phenotypically normal larvae at 5dpf are het/wt. | 6dpf: heart edema, small eyes, eye edema; n =25/25 with phenotype are mutant |
