# Supplementary figures and images for: Mitochondrial proteins encoded by the 22q11.2 neurodevelopmental locus regulate neural stem and progenitor cell proliferation
Source: Mol Psychiatry. Author manuscript; Available in PMC 2024 Feb 16. (PMC10730408; doi:10.1038/s41380-023-02272-z)

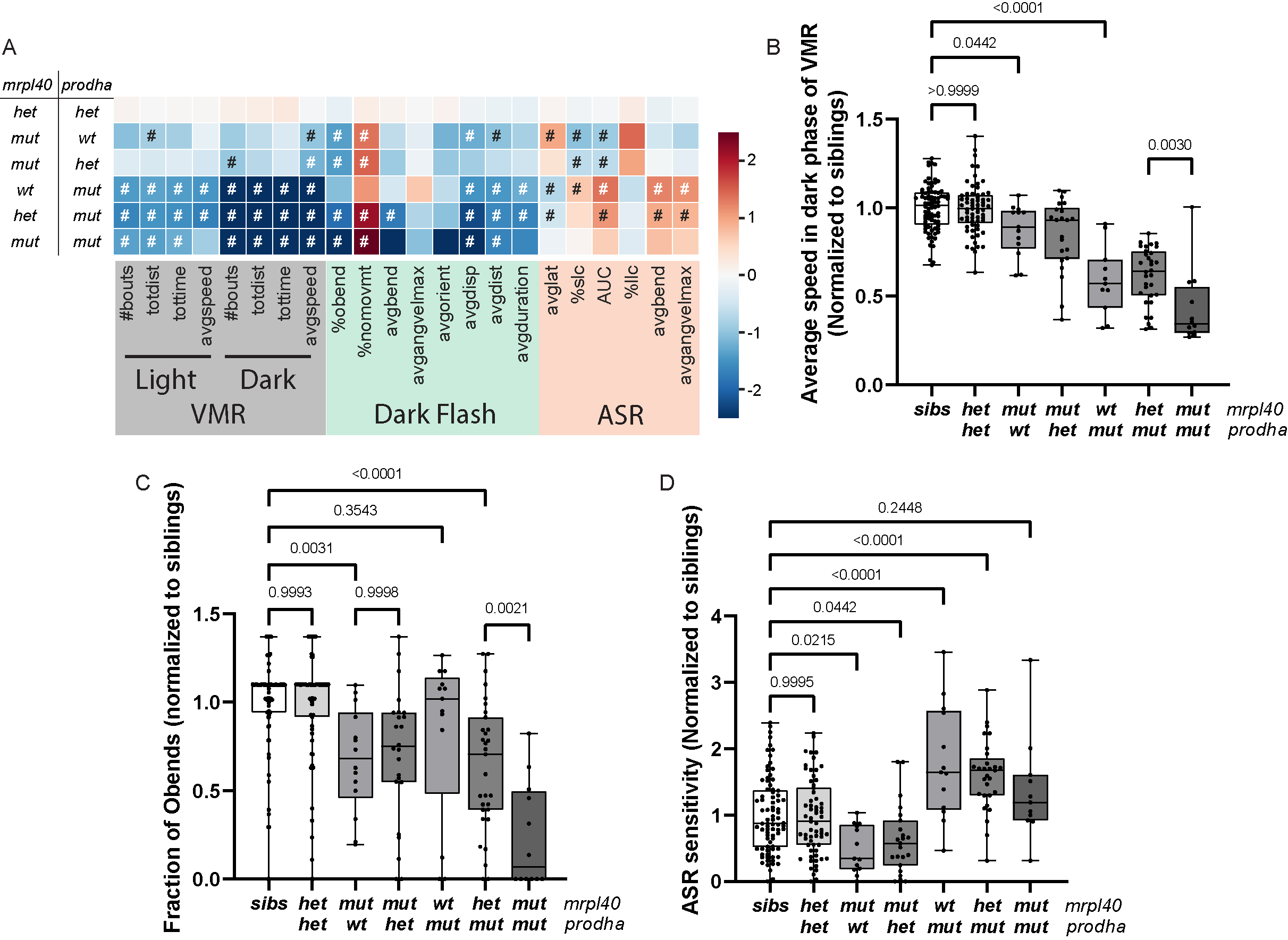

Supplement: Supplemental Figure 4 [file NIHMS1950658-supplement-Supplemental_Figure_4.tif]

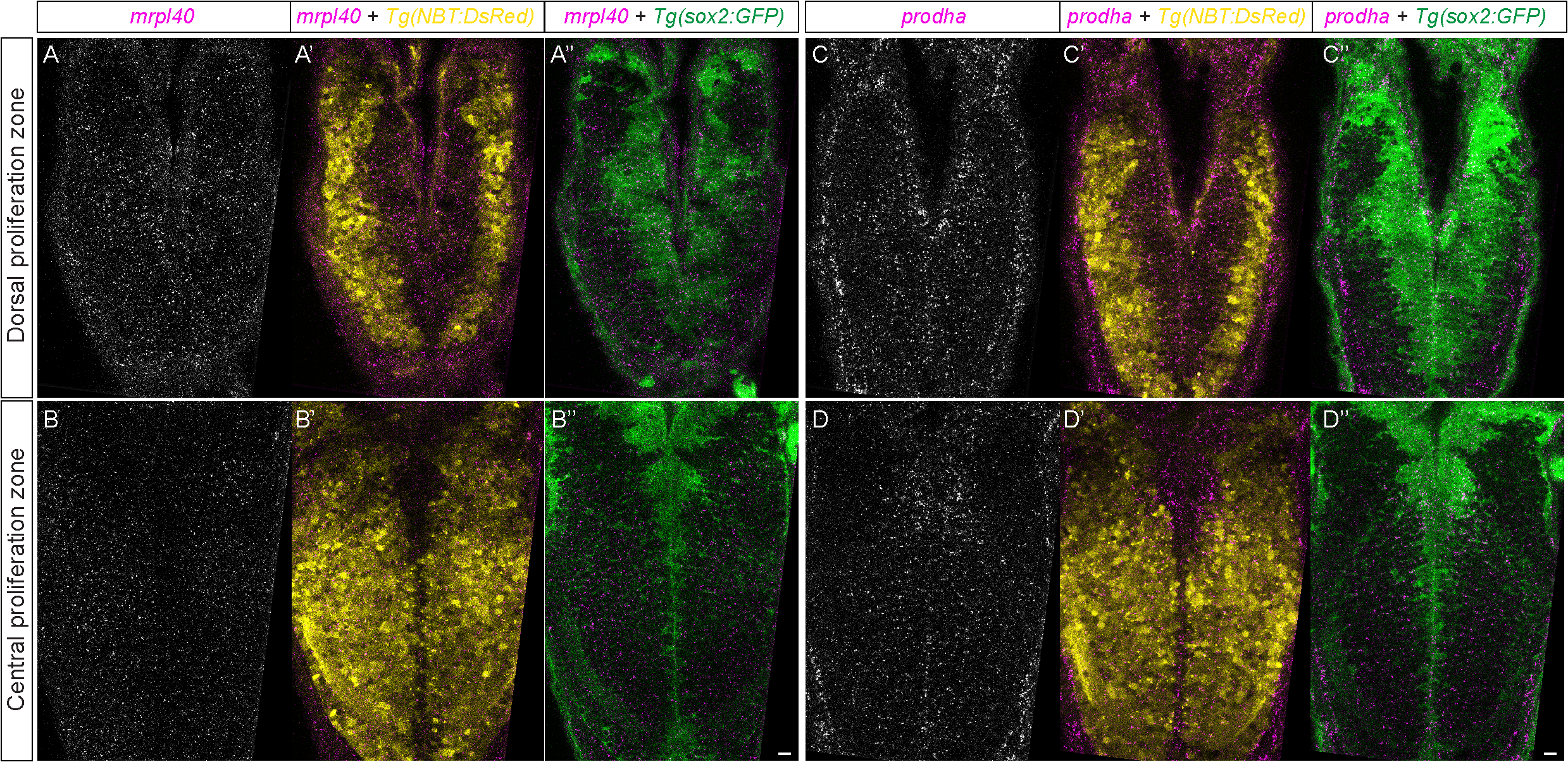

Supplement: Supplemental Figure 3 [file NIHMS1950658-supplement-Supplemental_Figure_3.tif]

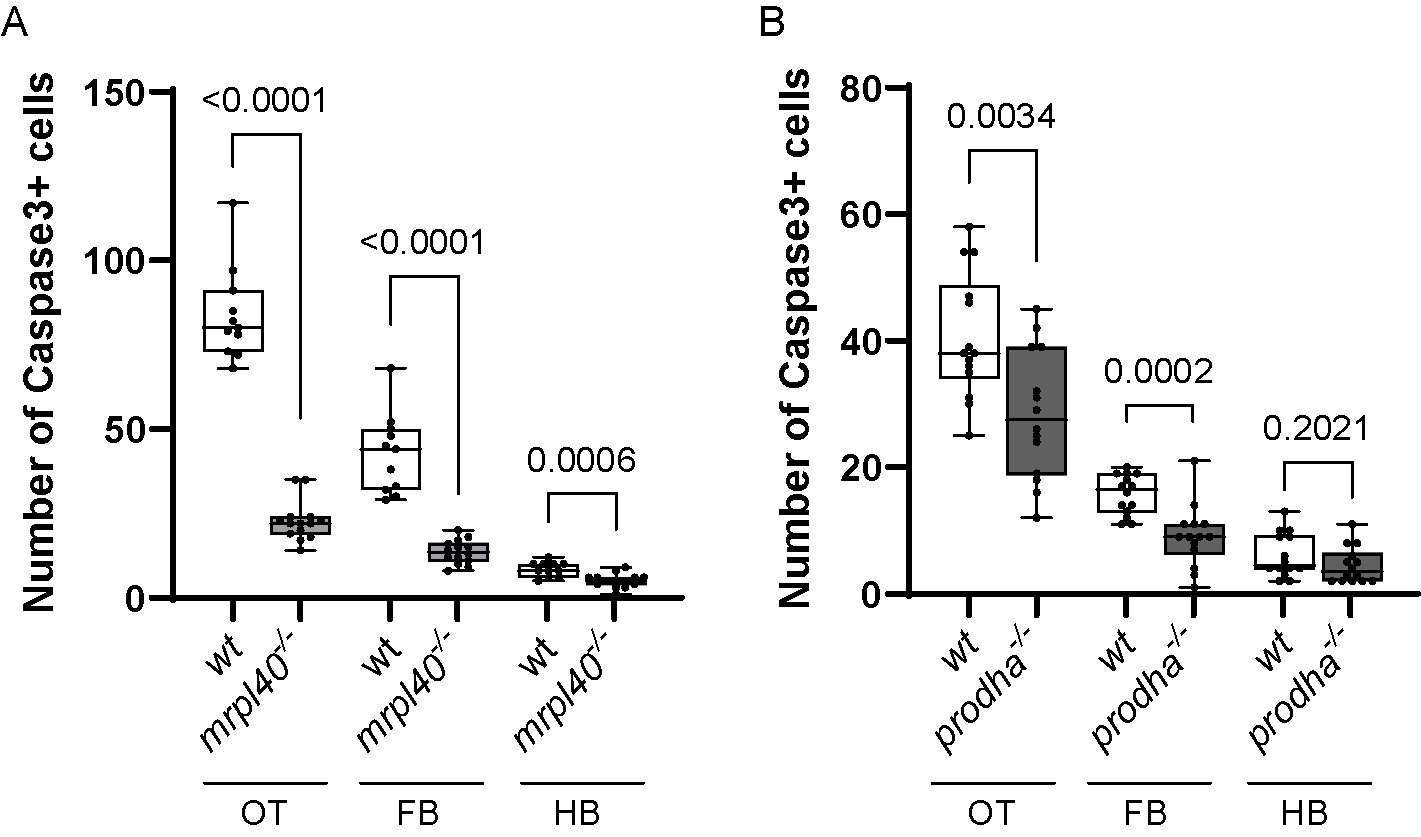

Supplement: Supplemental Figure 2 [file NIHMS1950658-supplement-Supplemental_Figure_2.tif]

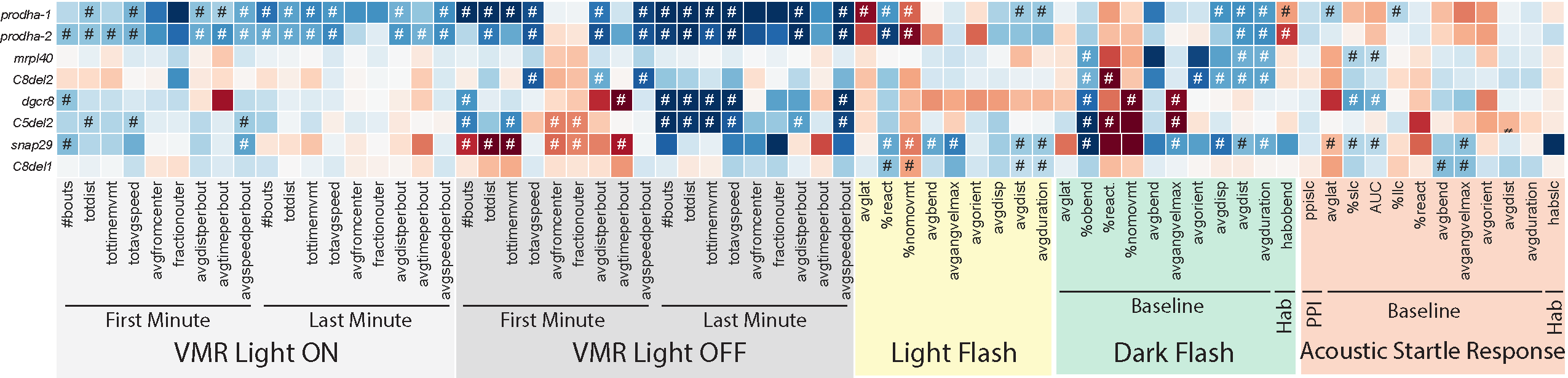

Supplement: Supplemental Figure 1 [file NIHMS1950658-supplement-Supplemental_Figure_1.tif]
