# Supplementary material for: Mitochondrial proteins encoded by the 22q11.2 neurodevelopmental locus regulate neural stem and progenitor cell proliferation
Source: Mol Psychiatry. Author manuscript; Available in PMC 2024 Feb 16. (PMC10730408; doi:10.1038/s41380-023-02272-z)
Supplement: Supplemental Table and File titles and legends [file NIHMS1950658-supplement-Supplemental_Table_and_File_titles_and_legends.docx]

**Supplemental Information**

**Supplemental Figure 1.** Single gene mutants are phenocopied by second allele disrupting same gene

Heatmap illustrating mutant phenotypes across behavioral metrics. Each row is an individual mutant line and all columns represent a behavioral metric. Color of each box represents difference of average mutant value compared to siblings expressed as number of standard deviations (SD). Blue boxes indicate the value is lower in the mutant and Red boxes indicate the value is higher in the mutant. Mutant values that are significantly different from siblings based on Student’s t-test with a Bonferroni-corrected p-value of 0.05/94 are designed with #. VMR behavioral metrics are highlighted in gray, LF in yellow, DF in green, and ASR in orange. Two independent single gene *prodha* mutant lines display the same phenotype across behavioral metrics. Combination gene lines *C8del2*, *C5del2*, and *C8del1* that overlap *mrpl40*, *dgcr8*, and *snap29* respectively, phenocopy single gene lines, indicating single genes are the drivers of the combination line phenotypes.

**Supplemental Figure 2.** *mrpl40* and *prodha* mutants do not display increased apoptosis

(A) Caspase-3 antibody staining quantification for *mrpl40* mutants at 5dpf. In all brain regions quantified, *mrpl40* mutants have fewer apoptotic cells than wildtypes.

(B) Caspase-3 antibody staining quantification for *prodha* mutants at 5dpf. In the optic tectum and forebrain, *prodha* mutants have fewer apoptotic cells than wildtypes.

Unpaired two-way Student’s t-tests, p-values displayed on the plots. OT, optic tectum; FB, forebrain; HB, hindbrain.

**Supplemental Figure 3.** *mrpl40* and *prodha* are expressed in hindbrain proliferative zones.

Hybridization Chain reaction *in situs* for *mrpl40* (A-B’’) and *prodha* (C’D’’) in wildtype larvae at 5dpf. Both *mrpl40* and *prodha* transcripts colocalize with the NSPC transgenic marker *Tg(sox2:GFP)* in the central and dorsal proliferative zones. Note that *prodha* appears to be enriched in the central proliferative zone. *Tg(NBT:DsRed)* labels post-mitotic neurons.

**Supplemental Figure 4.** *mrpl40* and *prodha* function independently from each other to regulate behavior

*(A)* Heatmap illustrating behavioral phenotypes across behavioral metrics, as described for Figure S1. Values that are significantly different from siblings based on Student’s t-test with p-value of 0.01 are designed with #. Transheterozygotes do not have phenotypes across the behaviors assayed. Specific phenotypes are further displayed in Figures S4B-S4D.

(B) Average speed in dark phase of VMR. Double mutants have lower average speed than either single mutant alone.

(C) Fraction of O-bends in response to DF stimuli. Double mutants perform fewer O-bends than either single mutant alone.

(D) ASR sensitivity. Double mutants display a phenotype intermediate between *mrpl40* and *prodha* single mutants. One-way ANOVA, p-values are displayed on the plots.

**Supplemental Table 1**. Single gene mutations, gRNA sequences, and genotyping primer sequences (See excel spreadsheet)

**Supplemental Table 2.** Morphological phenotypes observed in homozygous mutants. Related to Figure 1.

**Supplemental Table 3.** Incompletely penetrant swim bladder inflation phenotypes observed. Related to Figure 1.

**Supplemental Table 4.** Combination gene mutations, gRNA sequences, and genotyping primer sequences (See excel spreadsheet)

**Supplemental Table 5.** Annotated example of behavioral output for single behavioral experiment (See excel spreadsheet)

**Supplemental Table 6.** Summary data for eight mutant lines with behavioral phenotypes. Two independent experiments performed for each line. Table denotes behavioral metrics where p<0.05 in both experiments (See excel spreadsheet)

**Supplemental Table 7.** Normalized and pooled data for each of the eight mutant lines with behavioral phenotypes. p values also included for each behavioral metric. (See excel spreadsheet)

**Supplemental file 1.** Python script used for ASR behavioral tracking. (See Python file)

**Supplemental file 2.** Python script used for DF behavioral tracking. (See Python file)

**Supplemental file 3.** Python script used for LF behavioral tracking. (See Python file)

**Supplemental file 4.** Python script used for VMR behavioral tracking. (See Python file)
